# Ultra-slow mechanical stimulation of olfactory epithelium modulates consciousness by slowing cerebral rhythms in humans

**DOI:** 10.1038/s41598-018-24924-9

**Published:** 2018-04-26

**Authors:** A. Piarulli, A. Zaccaro, M. Laurino, D. Menicucci, A. De Vito, L. Bruschini, S. Berrettini, M. Bergamasco, S. Laureys, A. Gemignani

**Affiliations:** 1 Department of Surgical, Medical, Molecular and Critical Area Pathology, University of Pisa, Via Roma 65, 56126 Pisa, Italy; 2 Coma Science Group, GIGA Research Center, University and University Hospital of Liège, Avenue de l’Hôpital 11, 4000 Liège, Belgium; 3 Institute of Clinical Physiology, National Research Council (CNR), Via Giuseppe Moruzzi 1, 56127 Pisa, Italy; 4 Department of Translational Research and New Technologies in Medicine and Surgery, University of Pisa, Via Risorgimento 36, 56126 Pisa, Italy; 5 Azienda Ospedaliero-Universitaria Pisana (University Hospital, AOUP), Via Paradisa 2, 56124 Pisa, Italy; 6 PERCRO Laboratory, TECIP Institute, Sant’Anna School of Advanced Studies, Via Alamanni 13B, 56010 Ghezzano Pisa, Italy

## Abstract

The coupling between respiration and neural activity within olfactory areas and hippocampus has recently been unambiguously demonstrated, its neurophysiological basis sustained by the well-assessed mechanical sensitivity of the olfactory epithelium. We herein hypothesize that this coupling reverberates to the whole brain, possibly modulating the subject’s behavior and state of consciousness. The olfactory epithelium of 12 healthy subjects was stimulated with periodical odorless air-delivery (frequency 0.05 Hz, 8 s on, 12 off). Cortical electrical activity (High Density-EEG) and perceived state of consciousness have been studied. The stimulation induced i) an enhancement of delta-theta EEG activity over the whole cortex mainly involving the Limbic System and Default Mode Network structures, ii) a reversal of the overall information flow directionality from wake-like postero-anterior to NREM sleep-like antero-posterior, iii) the perception of having experienced an Altered State of Consciousness. These findings could shed further light via a neurophenomenological approach on the links between respiration, cerebral activity and subjective experience, suggesting a plausible neurophysiological basis for interpreting altered states of consciousness induced by respiration-based meditative practices.

## Introduction

In recent years, an amount of scientific literature has been devoted to the investigation of brain activity changes induced by meditation^1^. In this context, given the heterogeneity of meditative techniques, a large variety of findings have been described. Still, a result appears consistent among different studies: a widespread enhancement of EEG theta activity (4–8 Hz) and of its synchronization among multiple brain regions^1,2^. Both findings might be related to the marked slowing of nasal respiratory rhythm, which is a fundamental basis of many meditative techniques. As stated by Fontanini and Bower (2006)^3^, *“the influence on oscillatory brain states of the olfactory system in general, and of respiration, provides a potentially interesting new perspective on meditation”*.

This hypothesis has gained further credit after a study demonstrating that olfactory neurons of mammalians (including humans) beyond their role as odorants detectors, respond also to mechanical stimuli^4^. In 1942 Nobel Laureate Edgar Douglas Adrian^5^, did already observe that the hedgehog olfactory epithelium mechanical stimulation produced a discharge of impulses in its olfactory bulb depending on the airflow pressure. Years later, it was demonstrated that respiration-related airflow on the frog olfactory mucosa synchronized its cortical EEG activity, probably via axo-dendritic connections starting from the olfactory bulb^6^. Since those years, while the role of nasal breathing as a co-factor facilitating olfactory processing has been extensively investigated^7,8^, research on the role of non-olfactory functions of the olfactory epithelium has been largely neglected until recent years.

A review article^3^ highlighted, in animal models, a parallelism between olfactory bulb and thalamus in driving slow oscillatory patterns (0.3–1 Hz) of cortical neurons (i.e. Slow Oscillations, consisting of an alternation between electrical silence and synaptic activity) both during Slow Wave Sleep and ketamine-xylazine anesthesia. The authors showed that the rhythmic input of odorless air in the nostrils triggered a neuronal oscillatory pattern characterized by the alternation between depolarization and hyperpolarization in the olfactory cortex. Notably, this pattern was disrupted after tracheotomy. These data indicate that a sensory signal driven by the periodic entry of air into the nose modulates the emergence of slow-wave synchronization in the olfactory system.

They hypothesized that, given the large number of connections between the olfactory system and the prefrontal cortex^9,10^, the Slow Oscillations could propagate further to the prefrontal cortex from where they would invade the entire cortical mantle. A recent study^11^ contested this hypothesis, demonstrating that respiratory-related oscillatory neural activities are only detectable in primary olfactory structures with no global entrainment, at least when considering anesthesia or Slow Wave Sleep; on the contrary, the study evidenced a significant coupling between respiratory activity and non-olfactory cortical structures (neocortex and hippocampus) during anesthesia-induced REM-like states. Similarly to the latter state, a study on the activity of whisker barrel cortex in awake mice^12^, found that oscillations within delta band (0.5–4 Hz) as well as gamma band (30–80 Hz) amplitudes, are phase locked to respiration. Notably, the removal of the olfactory bulb eliminated both cortical entrainments.

It was recently demonstrated both in the animal model^13^ and in humans^14^, that the synchronization of cortical and sub-cortical structures driven by nasal respiration via olfactory bulb is mainly linked to the inspiratory phase of the respiratory cycle. In the context of breathing physiology, inspiration can thus be considered the main trigger of neural entrainment, and could acquire a particular importance since it is the respiratory phase mainly under volitional and cognitive control^15^.

Coming back to meditation, an unavoidable confounding factor when investigating the role of respiration in modulating brain activity is that slow-paced respiration is typically coupled to attention at large or even focused attention,  a condition which in turn activates specific cortical networks^16^.

Is it possible to study in humans the mechanical effects of the inspiratory phase of slow-paced breathing on the olfactory epithelium, olfactory bulb and cortical electrical activity controlling for stimulation features such as air-pressure, duration and targeted area of the nasal vault, avoiding at the same time cognitive efforts, cardio-respiratory reflexes and specific postures associated with meditation?

Starting from these considerations and hypotheses, we herein demonstrate that a slow-paced and focused stimulation (0.05 Hz) of the olfactory epithelium through periodically delivered (cycle length 20 s, continuous odorless air delivery 8 s) odorless air at a perceivable pressure has the following effects:

1. An increase of slow brain rhythms (delta and theta) over the whole cortical mantle, including structures pertaining to the olfactory system, parahippocampal gyrus, orbitofrontal cortex and areas belonging to the Default Mode Network (DMN)^17^.
2. A reversal of theta band information flow directionality between selected cortical hubs from wake-like postero-anterior to Slow Wave Sleep-like antero-posterior^18^.
3. Changes described in the previous points are associated to modifications in the subjective perception of the self together with a shift in the direction of attentional resources towards the self, a subjective sensation of being in an altered state of consciousness coupled to a significant reduction in the ability of rational thinking and thoughts control^19^.

## Results

We assessed the effects of a low frequency mechanical stimulation focally delivered to the olfactory epithelium (via nasal cannulae, 0.05 Hz, 8 s of continuous odorless air delivery, 12 s off; see Materials and Methods - ‘Overview’ and ‘Experimental procedures’ and Supplementary Material, Section A, SM-A) on the cortical electrical activity of fifteen healthy volunteers of matched age (7 females). Cortical activity was monitored by means of high-density EEG and breathing rhythms by a piezo-resistive belt placed on the abdomen. Each subject underwent two sessions administered in a randomized order at one-week one from the other: a nasal stimulation (NS) and a sham Control session (SC). Each session included a period of 7 min before the NS (SC), and a period of 7 min after (from now on identified as pre- and post-periods). After the session, a set of psychometric tests was administered to the subject, evaluating executive and hippocampal functions as well as state of consciousness: i) Digit Span Forward and Backward^20^, ii) Corsi block test^21^, and iii) Phenomenology of Consciounsess Inventory, PCI^19^. EEG-derived features and breathing rhythms related to the pre- and post- periods were collected both for the NS and the SC after a proper pre-processing (Materials and Methods - ‘Experimental procedures’ and ‘Signals pre-processing’). EEG features related to the post-NS were analyzed and compared both to the pre-NS and to the post-SC (SM-B), applying paired t-tests with Statistical Non-Parametrical Mapping approach for statistical images (SnPM)^22,23^, taking thus into account the multiple comparisons issue; the significance threshold was set at p < 0.05.

Owing to the pre-processing procedures, three volunteers were excluded from the analysis: one subject (female) was discarded as her EEG during the SC session was contaminated by massive movements and muscle artifacts, the other two (males) were excluded as they showed typical sleep EEG patterns during at least one of the two sessions (N1 and/or N2 sleep stages according to AASM criteria for sleep scoring^24^ EEG recordings were visually inspected by sleep EEG experts: AP, DM, AG). Twelve subjects (6 males and 6 females, age 22 ± 1.5, mean ± standard error), were retained for the analyses.

### The stimulation does not produce any change in mouth-breathing rhythms

Breathing rhythms of pre- and post- NS and SC were collected and submitted to a repeated measure ANOVA with *condition* (NS-SC) and *period* (pre-post) as two levels within-factors. No significant effect of *condition*, *period* or of their interaction was apparent (see SM-C).

### The stimulation induces a widespread enhancement of delta and theta activity

**Figure 1:**
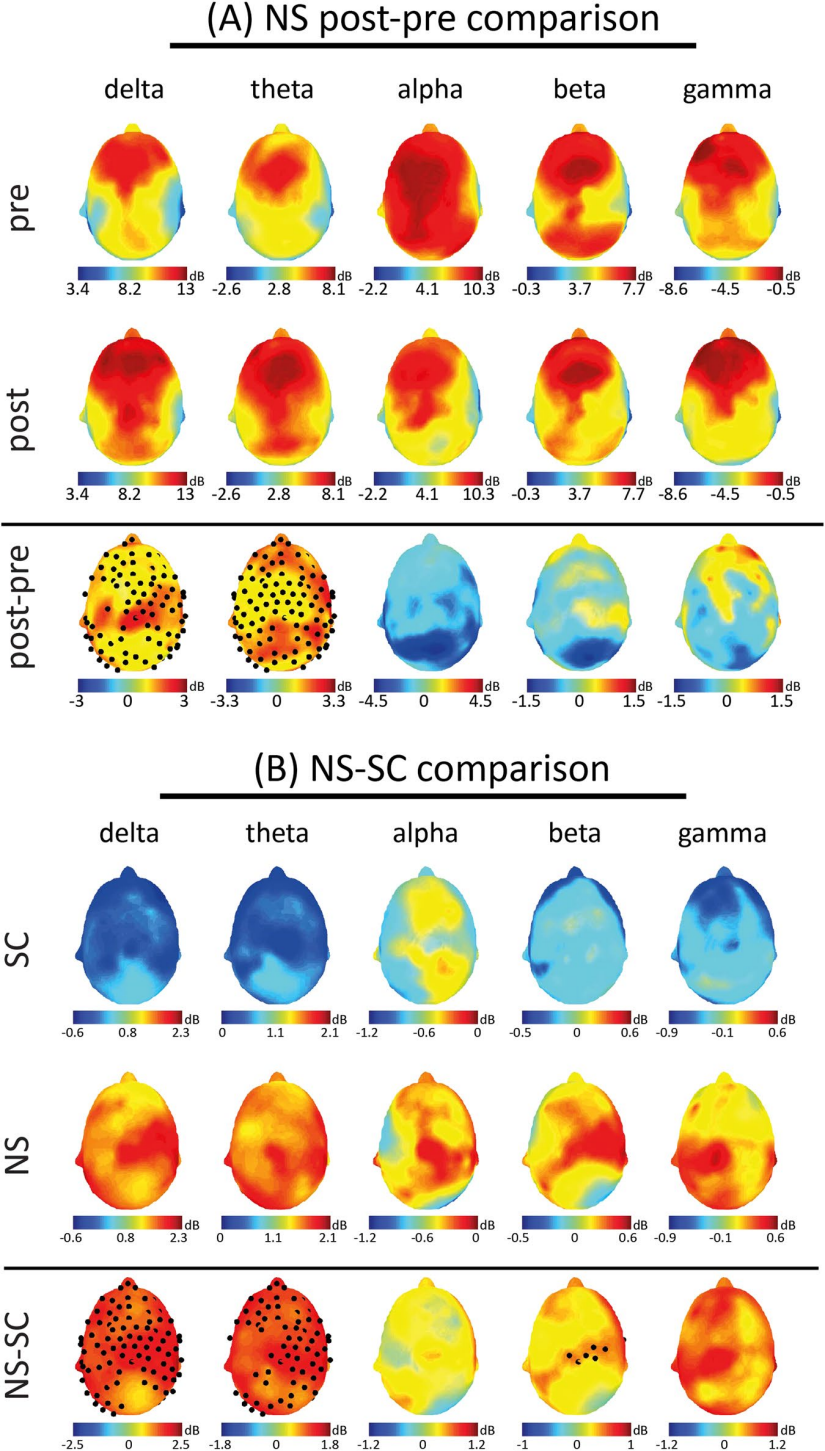
(**A**) Topographical distributions of mean (among subjects) log-transformed power in the five bands of interest are presented for the NS. First row refers to the pre-period, second row to the post- and third row to the comparison between subjects’ post- and pre-periods. Note that for pre- and post-NS maps the same scale is used to ease the reader in the comparisons’ interpretation. (**B**) Topographical distributions of mean (among subjects) normalized power in the five bands of interest are presented for post-SC and post-NS periods. Log-transformed powers of the post- periods are normalized to their respective log- transformed pre- period values (ratio between post- and pre-periods values). First row refers to post-SC, second row to post-NS and third row to the comparison between subjects’ post- NS and post-SC. Note that for post-SC and post-NS maps the same scale is used to facilitate the reader in the comparisons’ interpretation. Throughout the figure, electrodes showing significant (at least p < 0.05) power increases are highlighted by black dots; no significant decrease was observed for any comparison.

The average EEG power density of each channel was estimated both for the NS and SC sessions in five bands of interest (delta, 1–4 Hz, theta, 4–8 Hz, alpha 8–12 Hz, beta, 12–30 Hz, gamma 30–40 Hz). Subjects’ post-NS power densities were compared to their corresponding pre-NS values: a massive increase of both delta and theta band power was observed after the NS. As apparent from Fig. 1A, significant increases were detectable in the post-period as compared to the pre- on fronto-central, left-temporal and occipital areas for delta, while for theta the increase was significant over the whole scalp. At variance, the SC did not induce any significant change for any of the considered bands (SM-D).

For each session and band, the channel power related to the post-period was divided by its corresponding pre- value. The comparison between subjects’ post-NS and post-SC yielded results similar to those related to the NS post-pre comparison (Fig. 1B; SM-B for statistical analyses and normalization rationale). An increase of power for both delta and theta was observed: delta band increases were significant over the whole cortex; when considering theta band, significant increases were apparent within fronto-central, posterior-occipital and right centro-temporal areas. A significant power enhancement (six electrodes of the central area), was found also for beta band.

**Figure 2:**
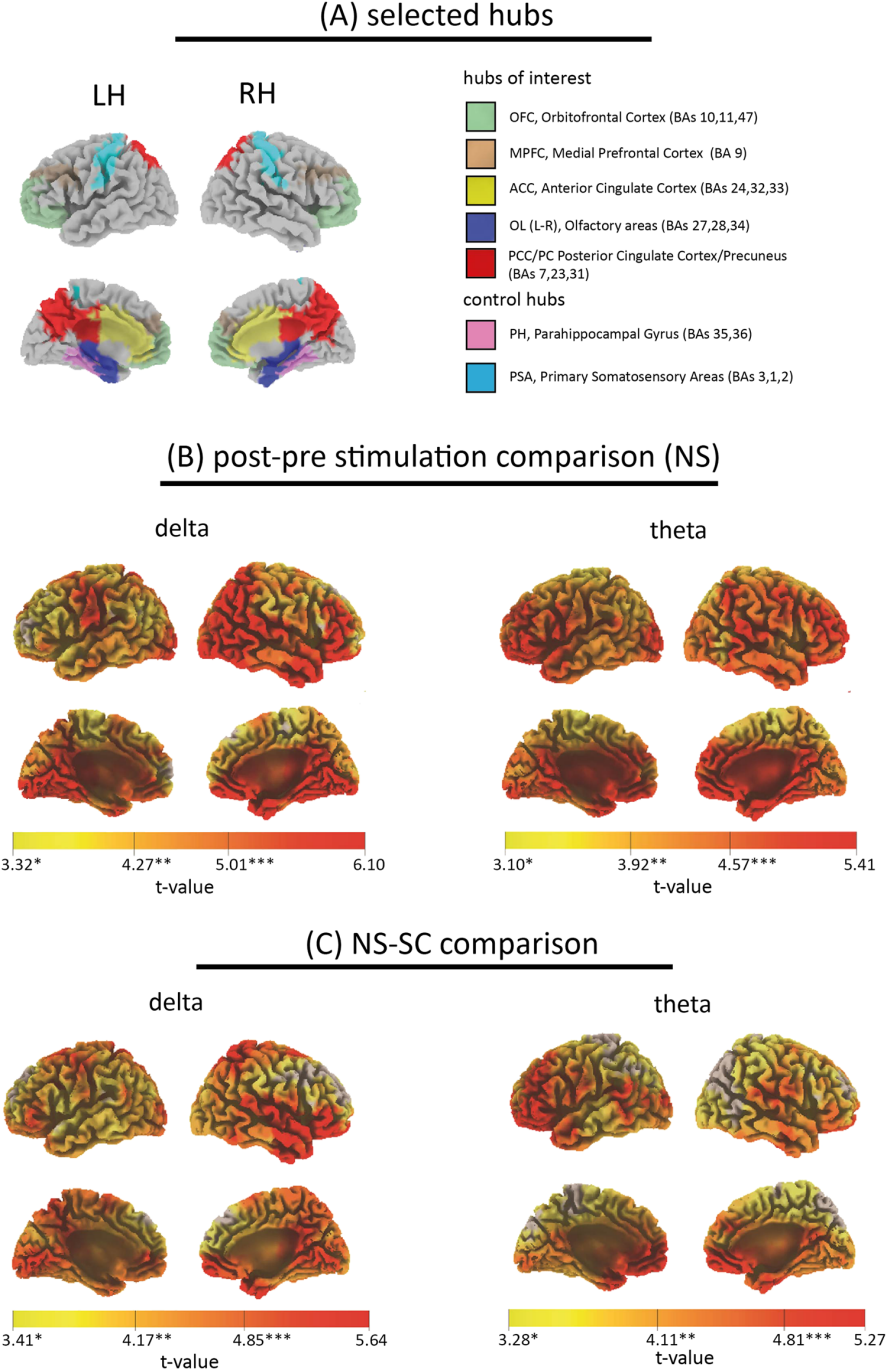
(**A**) A synopsis of the selected cortical hubs of interest is presented as a guide for the interpretation of results obtained from sLORETA and Granger Causality analyses. **(B**) Voxel t-values distributions obtained in the statistical comparison between post- and pre- NS are presented for delta (left panel), and theta (right panel). **(C)** Voxel t-values distributions obtained in the statistical comparison between post-NS and post-SC are presented for delta (left panel) and theta (right panel). Throughout the images, only significant voxels (p < 0.05 after SnPM correction) are represented (non-significant voxels are left uncolored). Colors range between yellow (p < 0.05) and red, the latter denoting the highest t-value among all significant voxels in the statistical image. For each band and comparison, t-values thresholds for p < 0.05, p < 0.01 and p < 0.001 are highlighted on the color bar by one, two or three asterisks respectively.

The band-wise comparisons performed at the scalp level were replicated at the cortical level, estimating source spectral densities by means of sLORETA in the frequency domain^25,26^ for delta and theta bands. A massive significant increase in spectral content within both bands was found during the post-NS as compared to the pre-NS period, in agreement with findings of scalp power spectral analysis. Relevant increases were found within orbitofrontal cortex (OFC), medial prefrontal cortex (mPFC, bilaterally for theta band and with a right hemispheric prevalence for delta band), parahippocampal gyrus, enthorinal and cingulate cortices and right temporal lobe (Fig. 2B). At variance, no significant difference was found between post- and pre-SC. Relevant increases were apparent for the OFC (bilaterally but with a right hemispheric prevalence for delta and a left one for theta band) when comparing the post-NS to the post-SC (post- values were divided by their corresponding pre- values). An enhancement of spectral density within the mPFC (left hemisphere), was found for theta band, while an increase of the cingulate cortex sources spectral density was apparent for both bands (prominently in the posterior cingulate cortex, PCC); the precuneus (PC), showed an enhancement of source power density when considering delta band. An overall increase in the parahippocampal gyrus power density was found for both bands: the most relevant activations were found within the uncus and the rostral section of the gyrus. A further significant enhancement (delta band) was identified in the right temporal lobe (Fig. 2C).

### NS effects on cortical hubs of interest are significantly higher than those observed for the primary somatosensory areas

**Figure 3:**
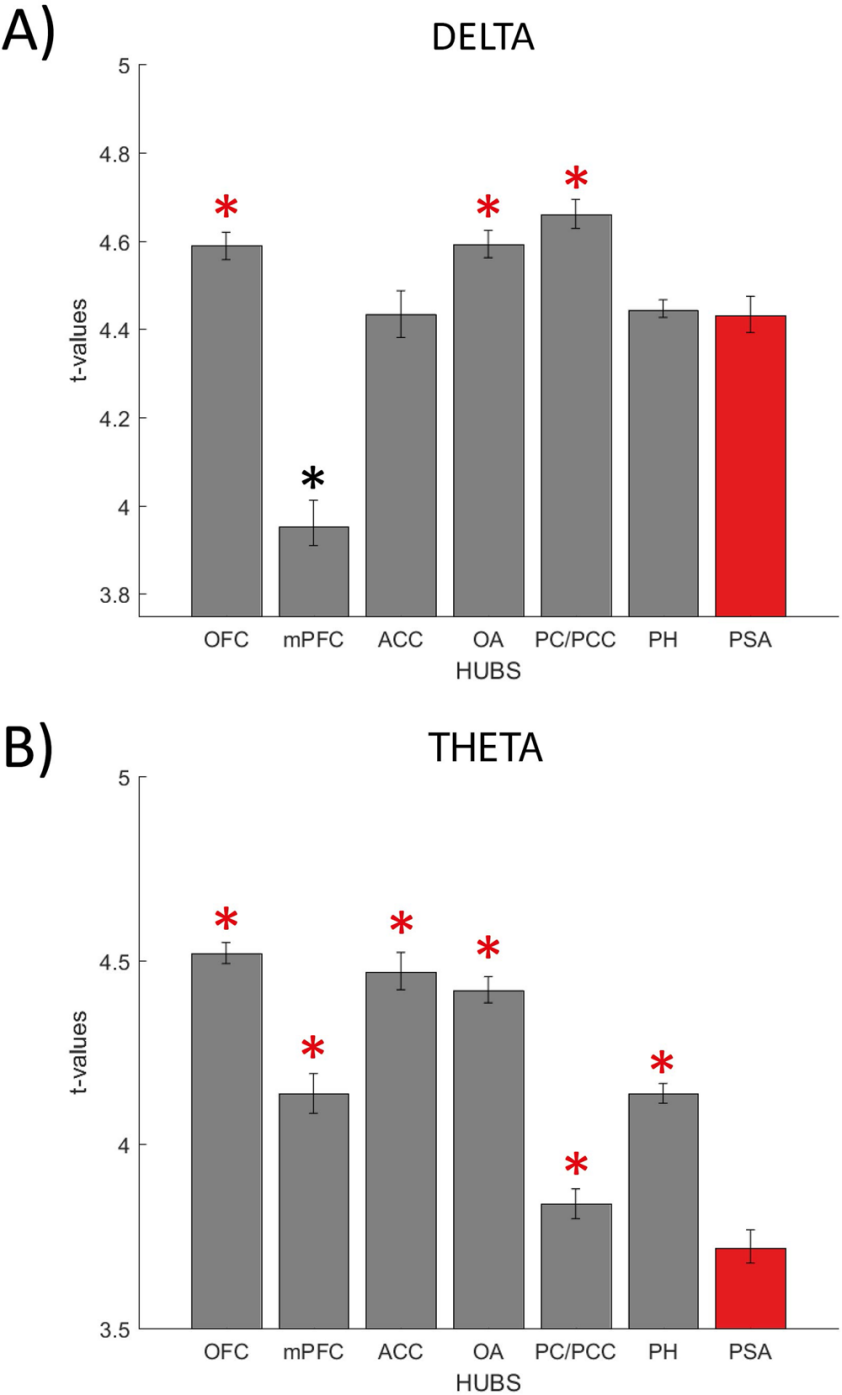
Descriptive statistics (mean ± standard error) of t-values related to the post-NS versus post-SC comparison in delta (panel A) and theta (panel B) bands are presented for eight selected cortical hubs. For each hub, the mean t-value and its standard error were estimated considering only the hub’s significant voxels (in the post-NS versus post-SC comparison). For both bands a significant *hub*-effect was found (p < 0.001, one-way ANOVA with *hub* as a between-factor). Planned post-hocs (each other hub versus PSA) were conducted. Red asterisks highlight hubs showing significantly (p < 0.001) higher values as compared to PSA, black ones those showing the opposite relationship (p < 0.001). Note that PH stands for parahippocampal gyrus.

Based both on our working hypothesis and on previous literature about relationships between breathing and brain activity, eight cortical hubs were selected: OFC, mPFC, anterior cingulate cortex, ACC, left and right olfactory areas (L-R OAs, each including the uncus, the rostral section of the parahippocampal gyrus and the enthorinal cortex), posterior cingulate cortex-precuneus, PCC/PC, parahippocampal gyrus, and primary somatosensory areas (PSA, Brodmann areas, BAs 3,1,2) which was chosen as a control hub. For each hub and band, t-values related to voxels showing a significant post-NS/post-SC difference were collected. The t-values series was submitted to a one-way ANOVA with *hubs* as a between-factor (Materials and Methods - ‘Cortical hubs selection and evaluation’ and SM-EF). A significant (p < 0.001) *hubs*-effect was found both for delta and theta; planned post-hocs were conducted for each band: t-values related to the hubs of interest were compared to those of the PSA. OFC, OA and PCC/PC had significantly higher t-values as compared to PSA (p < 0.001 for all) while PSA t-value was significantly higher than that of mPFC (p < 0.001) when considering delta band, whereas for theta all the hubs had significantly higher t-values with respect to PSA (p < 0.001 for all, Fig. 3 and SM-F).

### The stimulation promotes an enhancement of information flow both for delta and theta bands

**Figure 4:**
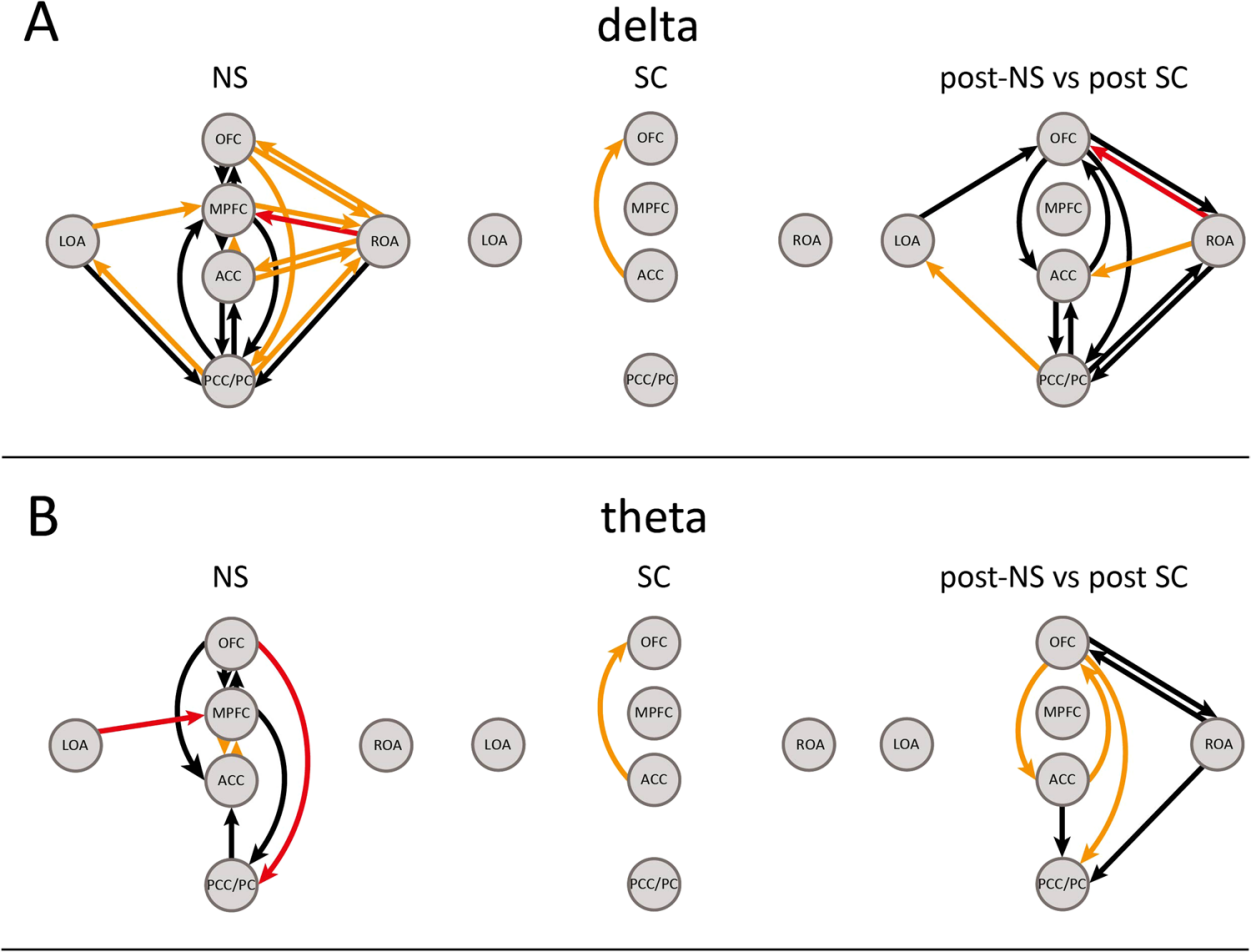
Significant differences in the directional information flow (GC) between cortical hubs related the post-/pre- periods and NS/SC comparisons are presented for delta in panel A, and for theta in panel B. Comparisons related to the NS are presented in the first column, those related to the SC are presented in the second column. Significant differences in the normalized directional information flow related to the post-NS versus post-SC comparison are presented in the third column. A colored arrow between hubs *i* and *j* denotes a significant increase of the information flow (nomalized information flow) in the *i* → *j* direction. Let *i* and *j* be two cortical hubs: the normalized information flow in the *i → j* direction was obtained for each session by dividing the post-period value by its corresponding pre-period value. Black, orange and red arrows respectively refer to significant increases at p < 0.05, p < 0.01 and p < 0.001.

Six cortical hubs among those mentioned in the previous sub-section, were retained for connectivity analyses based on our working hypothesis: OFC, mPFC, ACC, OAs (left and right) and PCC/PC. We verified whether the NS could promote changes in between-hubs flow of information. The standardized current density time course of each hub was obtained applying sLORETA in the time domain^26^ and averaging among the voxels pertaining to the hub (six time-series were obtained). Directional information flow between the selected hubs was estimated by applying Granger Causality (GC) in the frequency domain^27^ to the time series. A significant increase of bidirectional GC between most of the cortical hubs was found when comparing the post- to the pre-NS within delta band (Fig. 4A). Unidirectional increases of GC were found between OFC and PCC/PC and between left-OA and mPFC. For theta band, a bidirectional information flow increase was apparent both between the OFC and the mPFC and the mPFC and the ACC. Unidirectional GC increases involved the OFC on the one side and both ACC and PCC/PC on the other. Further unidirectional GC increases both between the mPFC and the PCC/PC and the left-OA and the mPFC were found (Fig. 4B). When considering the post- pre-SC comparison, the only significant enhancement was that between the ACC and the OFC for both bands (Fig. 4A,B, central column). Regarding the NS-SC comparison, information flow values related to post-periods were divided by their corresponding pre-periods values. Results related to delta band largely overlapped those obtained in the NS post-pre-comparison (Fig. 4A), whereas for theta significant bidirectional increases were found both between the OFC and the ACC and the OFC and the right-OA. Significant unidirectional increases were found between the OFC and the PCC/PC, between the right-OA and the PCC/PC as well as between the ACC and the PCC/PC (Fig. 4B).

For each hub, the net Causal Flow of Information (CFI, Materials and Methods - ‘Cortical hubs net Causal Flow of Information’) was then estimated as the sum of outcoming and incoming GCs (outcoming GCs were considered positive and incoming ones’ negative).

**Figure 5:**
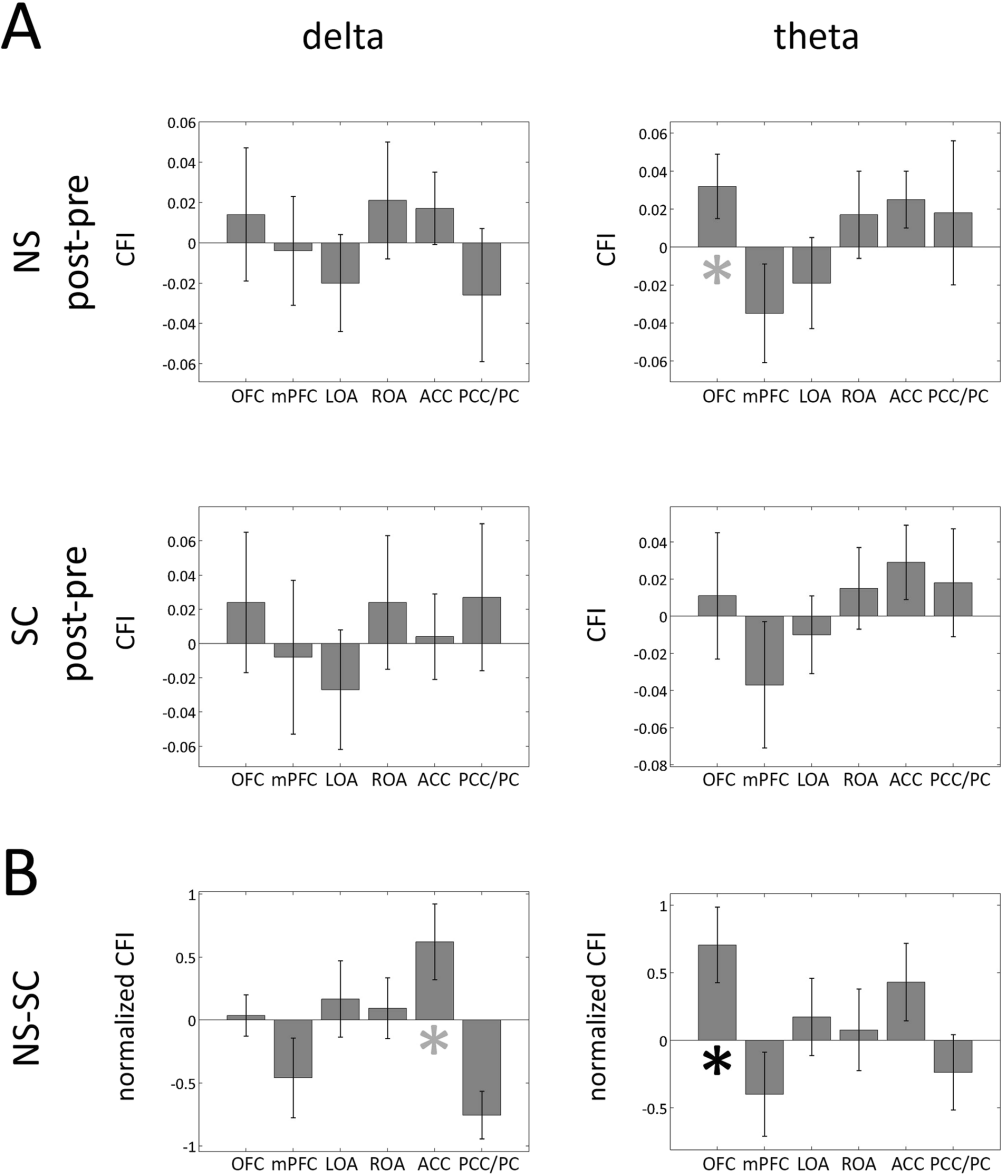
Comparisons in the net Causal Flow of Information (CFI), between post-NS (SC) and pre-NS (SC) are presented for each of the six hubs in panel A. First column refers to delta and second column to theta band. Comparisons related to the NS session are presented in the first row, and those related to the SC session in the second. A positive value of the net Causal Flow of Information corresponds to a prevalence of the outcoming flow with respect to the incoming flow. In Panel B comparisons related the normalized CFI between post-NS and post-SC sessions for each of the selected hubs are presented. In each graph and for each hub, the difference between post- and pre- or between post-NS and post-SC is presented: the bar represents the mean difference between the two periods’ values and the error-bar the mean ± standard error range. Gray asterisks denote a tendency towards significance (p < 0.1) whereas black ones’ significance at p < 0.05.

No significant difference in the CFI between post- and pre- periods was found either for the NS or the SC sessions both for delta and theta. A trend towards significance was found for the OFC in theta band (p < 0.08), with an enhancement of outcoming information flow during the post- as compared to the pre-NS, (Fig. 5A, first row, second column). A tendency towards significance was found for the ACC in delta band when comparing post-NS and post-SC (p < 0.08, higher outcoming causal flow of information for the post-NS as compared to the post-SC, Fig. 5B; details about CFI normalization in Materials and Methods - ‘Cortical hubs net Causal Flow of Information’). A significant increase (p < 0.04) was apparent for the OFC in theta band, with a higher outcoming normalized CFI after the NS (as compared to post-SC, Fig. 5B). As a further confirmation of these results, distributions of CFI in the four periods (pre-SC, post-SC, pre-NS, post-NS), both for the ACC in delta band and the OFC in theta, were tested against the null-hypothesis of being distributions with zero mean (i.e. not having a significant directionality): for each distribution, the 95% confidence interval of the mean value was estimated based on 1000 bootstraps derived from the original distribution. Anterior cingulate cortex CFI within delta band resulted significantly higher than zero (p < 0.05) in the post-NS, whereas the CFI of the orbitofrontal cortex within theta band was lower than zero in the SC session and significantly higher than zero in the post-NS period (p < 0.05, SM-G).

### The stimulation induces the perception of being in an altered state of consciousness

The comparison between NS and SC sessions highlighted differences in the subjective perception of specific dimensions of consciousness as indicated by Phenomenology of Consciousness Inventory items^19^. The NS as compared to the SC induced:

- Significantly higher scores in the Altered Experience (p < 0.03), in the Altered State of Awareness scale (p < 0.001) and in the Attention scale (p < 0.03), which, given the experimental condition and the results obtained from one of the related sub-scales (i.e. Direction of Attention, data not shown) were mostly referred to inward-directed Attention.
- Significantly lower Rationality and Volitional Control scores (p < 0.02 and p < 0.03 respectively).

**Figure 6:**
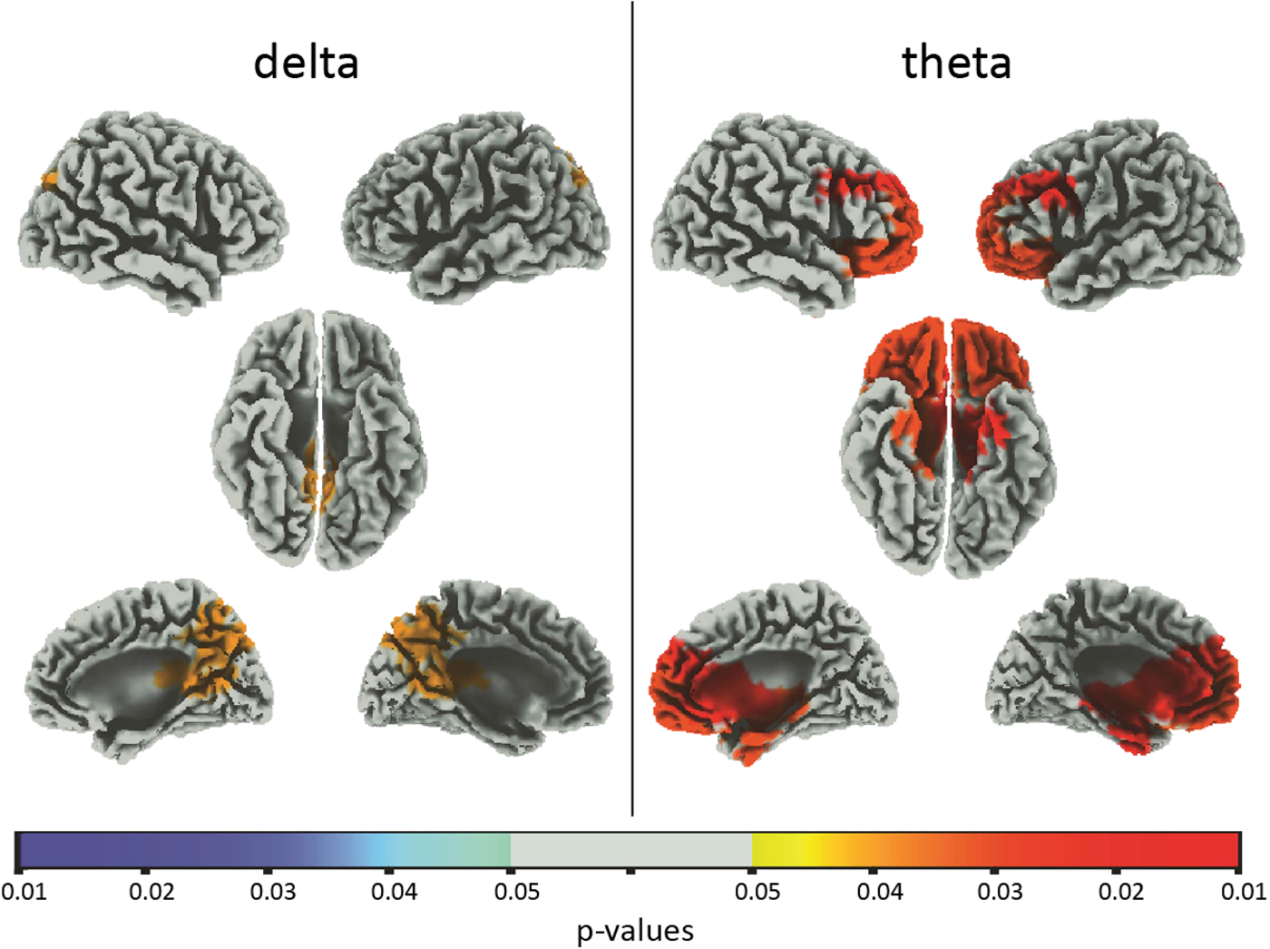
Significant correlations between increases in the subjective perception of having experienced an Altered State of Awareness (NS-SC comparison) and increases in the selected hubs power densities within delta and theta bands are presented. As apparent from the figure, the increase in the perception of having experienced an altered state of awareness is paralleled by: i) an enhancement of PCC/PC activity within delta band (p < 0.03) and ii) a concurrent increase in OFC, mPFC, and OAs electrical activity within theta band (p < 0.01).

At variance with PCI items, no significant difference in working memory-related functions was found (see Table 1 and Materials and Methods - ‘Psychometric assessment’). Increases in the subjective perception of having experienced an Altered State of Awareness (in the NS-SC comparison) were significantly correlated to increases in PCC/PC power density within delta band (p < 0.03) and concurrent increases in OFC, mPFC, and OAs power densities within theta band (p < 0.01), see Fig. 6 and Materials and Methods – ‘Correlations between psychometric differences (NS versus SC) and differences in cortical hubs activation’.

**Table 1:** Descriptive statistics (mean ± standard error) are presented for the administered psychometric tests/scales. First column refers to SC whereas the second to NS session. In the third column t-values related to the paired t-tests are presented, in the fourth the related p-values, and in the last one the corrected﻿ p-values.

	NS	SC	t-value	p-value	p-value*
Corsi Test	6.64 ± 0.28	7.18 ± 0.30	−1.94	0.068	0.170
Digit Span Forward	7.18 ± 0.30	7.45 ± 0.28	−1.00	0.367	0.550
Digit Span Backward	6.73 ± 0.41	6.45 ± 0.47	0.61	0.571	0.659
PCI Altered Experience	**2.05 ± 0.29**	**1.04 ± 0.21**	**3.04**	**0.009**	**0.030**
PCI Positive Affect	0.88 ± 0.35	0.91 ± 0.28	−0.06	0.965	0.965
PCI Negative Affect	0.74 ± 0.26	0.47 ± 0.21	0.88	0.403	0.550
PCI Attention	**4.40 ± 0.22**	**3.44 ± 0.32**	**3.75**	**0.007**	**0**.**030**
PCI Imagery	2.80 ± 0.41	3.09 ± 0.37	−0.69	0.494	0.617
PCI Self-Awareness	3.64 ± 0.47	4.49 ± 0.39	−1.27	0.223	0.372
PCI Altered State of Awareness	**2.82 ± 0.43**	**0.63 ± 0.17**	**4.51**	**0**.**001**	**0**.**001**
PCI Arousal	1.55 ± 0.47	1.55 ± 0.35	0.00	0.935	0.965
PCI Rationality	**3.48 ± 0.34**	**4.54 ± 0.31**	**−4**.**66**	**0**.**002**	**0**.**015**
PCI Volitional Control	**2.89 ± 0.38**	**4.18 ± 0.32**	**−2**.**86**	**0**.**010**	**0**.**030**
PCI Memory	3.37 ± 0.42	4.36 ± 0.25	−1.60	0.144	0.270
PCI Internal Dialogue	3.77 ± 0.47	2.64 ± 0.52	1.72	0.111	0.238

## Discussion

High-Density EEG recordings were acquired and analyzed for twelve healthy volunteers undergoing an olfactory epithelium stimulation with odorless air at a frequency of 0.05 Hz: during each 20 s cycle, air was continuously delivered to the olfactory epithelium for the first 8 s at a constant pressure. The stimulation lasted 15 min and the air stimuli were conveyed to the olfactory epithelium of both nostrils by nasal cannulae. Each nostril was sealed by a plug provided with a pass-through hole for the cannula to prevent any involuntary entering of air in the nostril. In this manner subjects were constrained to breathe with their mouth (see SM-A).

The frequency and duration of the stimuli were chosen accordingly to respiratory rhythms of meditative practices^28,29^. At variance with meditation and normal breathing, the stimulus was conveyed only to the olfactory epithelium to maximize its putative efficacy, controlling for stimulation features such as air-pressure, duration and targeted area of the nasal vault and avoiding at the same time cognitive efforts, cardio-respiratory reflexes and specific postures associated with meditative practices^16^.

As anticipated in the Introduction, the aim of the study was that of driving brain electrical activity and modulating consciousness by delivering rhythmic pressurized-air stimulations to the olfactory epithelium, whose receptors have been shown to be sensitive also to mechanical stimuli^4^.

We analyzed the post-NS periods looking for after-effects of the stimulation as compared both to the pre-NS and the post-SC (SM-B).

As expected, no difference in breathing rhythms was found among the four periods (pre-SC, post-SC, pre-NS and post-NS) since the subjects were forced to breathe through their mouths and as such, respiration was not influenced either by NS or SC (SM-C).

We found that the NS induced a significant and widespread increase of delta and theta power with respect to both pre-NS and SC; at variance, no significant difference was found either for alpha, beta or gamma bands. The global increase of slow frequencies was confirmed at the cortical level (see Fig. 2A,B). Some cortical structures were specifically involved in this enhancement: a) the OFC and the mPFC, b) the parahippocampal gyrus, the uncus, and the entorhinal cortex, c) the anterior and posterior cingulate cortices (ACC and PCC), d) the precuneus (PC). Directional connectivity analyses were conducted between six hubs involving both the fronto-parietal network (mPFC, ACC and PCC/PC)^17^, areas directly related to the olfactory system (OAs, i.e. uncus, anterior parahippocampal gyrus, enthorinal cortex) and the OFC, in line with Fontanini & Bower^3^ hypothesis.

The NS elicited a widespread and bidirectional information flow increase between the selected hubs in delta band whereas for theta the increase showed a prevalent antero-posterior directionality, with the OFC playing a pivotal role (Figs 4–5) The NS-SC comparison yielded a similar and widespread enhancement, albeit with a less pronounced antero- posterior directionality. When considering theta activity, the OFC behaved like a ‘source’ since the NS induced an increase of OFC net Causal Flow of Information as compared both to the pre-NS and to the SC, (i.e. the net information flowing outwards was significantly increased by the NS, Fig. 5). These findings assume a special neurophenomenological interest when interpreted in the light of the stimulation effects experienced by subjects:^19^ the NS elicited an altered perception of the self and of the flowing of time, a high degree of inwardly-directed attention together with a diminished ability of controlling their own thoughts, which led to a general perception of being in an altered state of consciousness. The increased perception of being in an Altered State of Awareness was significantly correlated with higher spectral densities within PCC/PC in delta band and OFC, mPFC, ACC, and OAs in theta.

The appearance or/and enhancement of slow rhythms (delta-theta) has been shown to be a common EEG correlate of states of altered or lacking consciousness: slow (delta and mostly theta) activities were observed during and/or after relaxation practices^30^, meditation^2,31–34^ and hypnotic states^35^. Slow rhythms characterize also all conditions of lower or absent consciousness be they physiological (NREM sleep^36^) or pathological^37–41^ (such as epilepsy^41^, Minimally Conscious State^42^ and Vegetative State/Unresponsive Wakefulness Syndrome^43^). While the mechanisms leading to the slowing of brain rhythms are different across the above-mentioned conditions, the link between alterations of consciousness and slowing of EEG frequencies holds for all of them.

The cortically widespread enhancement of slow rhythms driven by NS, together with the enhancement of information flow between hubs pertaining to the olfactory system, fronto-parietal network and/or to limbic structures, are coherent with the peculiar organization of the olfactory system itself and its wide range of anatomical connections with other cortical regions and subcortical structures. The role of the OFC acting as a ‘source’ of information flowing towards other cortical hubs can be interpreted in the light of its robust wiring with the piriform cortex (which in turn receives direct afferents from the olfactory bulb) as the orbitofrontal and prefrontal cortices have both direct^9,10,44^ and indirect (mediated by the thalamus) connections with the piriform cortex: this latter structure projects to the medio-dorsal thalamic nucleus (MDTN)^45^ which on its side has widespread projections to the OFC, including the orbital and rectal gyri^46^. Ongur and Price^46^ showed also the existence of connections between the mPFC on the one side and both the MDTN and limbic structures (including the olfactory bulb, the parahippocampal gyrus, and the cingulate cortex) on the other. As shown in the monkey^47^, the enthorinal cortex receives direct projections from the olfactory bulb and has bidirectional connections with the parahippocampal gyrus. The precuneus has reciprocal cortico-cortical connections with the PCC, the prefrontal cortex including the mPFC, the ACC and bidirectional projections with the thalamus targeting mostly its dorsal section^48^.

Sobel and colleagues^49^ described in the human model a modulatory effect of odorless air sniffing on cortical and sub-cortical structures’ electrical activity. They hypothesized two possible mechanisms generating these effects: an involvement of trigeminal afferents or a mechanical sensitivity of the olfactory nasal epithelium, which would convey the mechanical information to the olfactory bulb. Should the former hypothesis be true, we would expect a relevant activation of primary somatosensory areas following the NS^50^, whereas a relevant activation of the parahippocampal gyrus would favor the latter hypothesis, given the well assessed relationship between respiration (and olfactory bulb) and hippocampal activity. Effective demonstrations of this coupling were recently provided in the animal model by Lockmann *et al*.)^51^, who identified a hippocampal slow rhythm (<1.5 Hz) driven by respiratory frequencies and by Liu *et al*.^13^ who observed a modulatory effect of respiration on hippocampal sharp-wave ripples. A coupling at slower frequencies (0.16–0.33 Hz) was recently described also in humans^14^: it was demonstrated that natural breathing synchronizes electrical activity in the piriform cortex, as well as in the amygdala and in the hippocampus.

As a proof of the olfactory epithelium involvement, we verified that in delta but especially in theta band, post-NS increases were significantly higher (p < 0.001) both in our hubs of interest (OFC, mPFC, ACC, OAs, PC/PCC) and in the parahippocampal gyrus as compared to those related to the primary somatosensory areas. It is worth underlying that the finding related to the parahippocampal gyrus fits nicely with current literature about respiration-hippocampus entrainment^13,14,51^.

Based on the findings here described, it is tempting to draw a parallel: Kaminski *et al*.^18^ demonstrated that, while wakefulness is characterized by a parieto-frontal flow of information, NREM sleep is characterized by a fronto-parietal information flow (full-band, i.e. 0–30 Hz). The olfactory epithelium stimulation did generate a peculiar condition: an enhancement of low-frequencies and an information flow with a prevalent fronto-parietal directionality in theta activity shared also by that found in full-band (0–30 Hz) during Slow Wave Sleep by Kaminsky and colleagues^18^.

The Altered State of Awareness experienced by the subjects and induced by the NS was paralleled by enhanced slow activities within nodes of the DMN, such as PCC/PC, ACC and mPFC, which fits with the higher inward-directed attention reported by the volunteers. On the contrary, idle states (i.e. eyes-closed resting-state) while inducing an activation of DMN^17^, are paralleled by the enhancement of EEG alpha activity^52^, whose main generator has been identified in the thalamus^53,54^. We here hypothesize that in our experimental conditions the thalamus could have been driven and paced by its afferents (i.e. olfactory bulb and piriform cortex, whose activity is in turn paced by the NS). This pacing would have generated a burst mode activity of MDTN matrix cells which in turn would have promoted a widespread cortical delta-theta activity instead of the expected alpha.

The burst mode has been classically associated to drowsiness and sleep, but recent studies in the animal model have strongly questioned this assumption, showing that thalamic bursts can occur also during wakefulness^55–58^. The wake-related thalamic bursts seem to play a key role in encoding sensory information^59^ and in activating cortical circuits^60^. We cannot exclude that the tonic mode/burst mode shift of MDTN thalamocortical cells possibly induced by NS, could have altered integrative functions sustained by the reentry at prefrontal regions of MDTN thalamocortical volleys. An indirect support to this hypothesis stems from studies of MDTN in humans: MDTN lesions were associated with disinhibited or exaggerated responses to objects and environmental cues^61^ and alterations in time sense^62,63^. Notably, the “symptoms” experienced by the subjects submitted to NS, are similar to those reported in patients with MDNT lesions, although transient and devoid of any clinical relevance. The peculiar state experienced by the subjects could also be related to the high electrical activity of the OFC within theta band: the off-periods characterizing theta activity^64^ could be responsible for a global disfacilitation of the structure, possibly causing a decline of its main functionalities, such as decision-making and cognitive-emotional information processing^65–67^.

**Figure 7:**
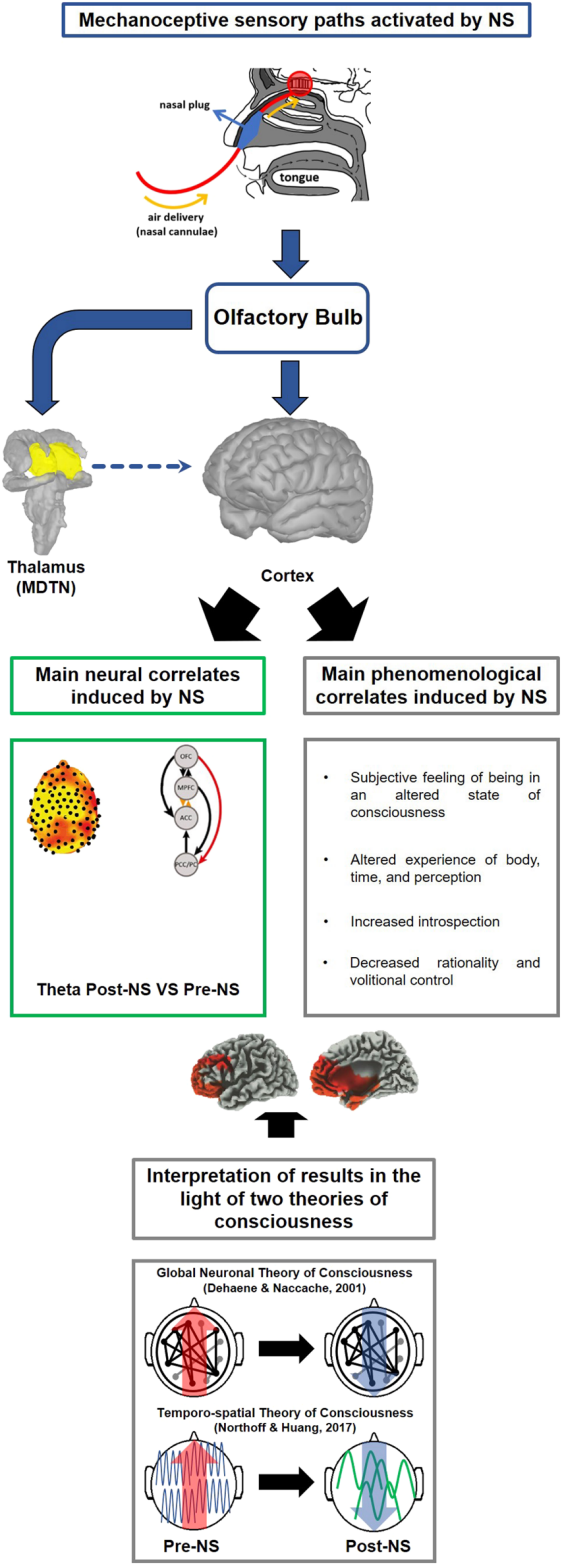
Synoptic representation of the experimental main stream from Nasal Stimulation (NS) to the theoretical interpretation of results in the context of Consciousness theories. From top to down, different aspects of the study are depicted: i) the NS and its effects on cortical and sub-cortical structures directly connected to the olfactory bulb (mechanoceptive olfactory paths); ii) main neural changes and phenomenological experiences induced by NS; iii) correlation between neurophysiological and phenomenological aspects (neuro-phenomenological domain); iv) heuristic interpretation of the experienced Altered State of Consciousness and of its neural correlates in the context of the Temporo-spatial Theory of Consciousness^68^ and of Global Neuronal Workspace Theory^69^ derived from Baars’ Global Workspace Theory^70^. In this last panel, changes in cortical rhythms and information flow directionality are highlighted. Details of each panel content are provided in the related manuscript section.

As a final consideration, the neurophysiological and behavioral results herein described can be interpreted in the context of the Temporo-spatial Theory of Consciousness^68^ and of the Global Neuronal Workspace Theory^69^ (Fig. 7). According to the former theory, nasal stimulation could have modified the subjects’ state of consciousness through the modulation of neural electrical activity as shown by the modification of EEG features both in temporal and spatial domains. The topologically widespread enhancement of slow rhythms (i.e. delta and theta) in wakefulness, together with the information flow modifications (mainly involving theta band) between hubs of interest (DMN and olfactory hubs), could represent the temporo-spatial phenomena underlying the subjective perception of being in an altered state of consciousness.At the same time, the Global Neuronal Workspace Theory^69^ derived from Baars’ Global Workspace Theory^70^, posits that different levels of consciousness are related to changes in the activation of ‘ignition’ thresholds belonging to thalamo-cortical and cortico-cortical networks needed by sensory stimuli for accessing consciousness. In our framework, the modulation of these thresholds (for instance due to a neuronal disfacilitation) could be the cause of the peculiar alterations of consciousness reported by subjects (i.e. altered perception of the body and of the self).In conclusion, the neurophysiological and phenomenological novelty of the effects of nasal stimulation on states of consciousness (Fig. 7) needs further investigation aiming at unveiling the complete scenario underlying the relationships between slow respiratory rhythms and consciousness: future lines of research will include functional MRI studies replicating our experimental paradigm, studies aiming at verifying whether the slow deep breathing typical of meditative practices induces effects similar to those observed in the present study by selectively blocking or allowing the airflow on the olfactory epithelium. Finally, according to a classical ‘Sherringtonian’ approach, studies using patients with selective anatomical lesions of the olfactory bulb or functional inhibition of olfactory epithelium (i.e. through local anesthesia) should be performed.

## Materials and Methods

### Overview

Fifteen healthy right-handed volunteers of matched age (7 females) were enrolled in the study. The study consisted of two experimental sessions administered in a randomized order at a distance of one week one from the other: a nasal stimulation session (NS) during which a stimulation of the olfactory epithelium with odorless air-delivery (frequency 0.05 Hz, cycle 20 s, continuous air stimulation 8 s) and a constant pressure of 1.1 bar, was delivered via nasal cannulae for 15 min; a sham control session (SC) with the same experimental setup and timing, during which no stimulation was delivered. The pressure of the delivered air was tuned to be easily perceivable and not unpleasant for the volunteers. Perceivability and pleasantness were validated on an ensemble of ten individuals who did not participate in the study. The employed cannulae had an inner diameter of 1.5 mm and were made of polyhexahydrotriazine (a polymeric material).

High-density EEG and respiratory signals were recorded before, during and after each session (NS or SC); at the end of the session a battery of psychometric tests was administered.

All subjects signed an informed consent; the study was approved by the University of Pisa Ethical Committee (AOUP ID 2805) and carried out in accordance with the tenets of the Declaration of Helsinki.

Inclusion criteria were:

- Age > 18 years
- No personal or family history of neurological or somatic disorders. Absence of psychiatric disorders was assessed by semi-structured interviews conducted by a senior psychiatrist (AG).
- Not having taken any drug acting directly or indirectly on the Central Nervous System in the previous six months.

All subjects were kept unaware of the aims of the study.

### Experimental procedures

All sessions started at 4 pm and were conducted at the Othorinolaringoiatry Unit of Cisanello Hospital (Pisa, Italy). Polygraphic recordings were carried-out using a Net Amps 300 system (GES300) and signals were acquired at 500 Hz sampling rate using the Net Station software (version 4.4.2); respiratory signals using a piezoresistive belt placed on the abdomen and EEG signals with a 128 electrodes HydroGel Geodesic Sensor Net (Electrical Geodesic Inc., Eugene, OR, USA). EEG channels were referenced to the vertex keeping impedance values below 50k Ω.

During each session subjects were lying on a bed in a darkened room, in a resting state condition (eyes-closed and ears-plugged). Before each session, an othorinolaringoiatrist inserted a nasal plug in each nostril to prevent the accidental entering of air from the environment. Each plug was provided with a pass-through hole for the nasal cannula; the opening of each cannula was positioned close to the olfactory epithelium of the nasal vault. Volunteers were thus forced to breath with their mouths (SM-A). The NS setup was exactly reproduced in the SC albeit no air stimulation was provided.

After setting up the polygraphic and the air-delivery systems, subjects were given 3 min to get comfortable with the set-up, and then signals were recorded for 30 min: 7 min during pre-NS period (SC), 15 min during NS (SC), and 7 min during post-NS(SC) period.

### Signals pre-processing

Pre- and post- stimulation (NS or SC) polygraphic signals, (EEG and breathing), were analyzed using Matlab (MathWorks, Natick, MA, USA); sLORETA free software^26^ was used for EEG cortical source analysis and Brainstorm Toolbox^71^ was used to generate exemplary images of the cortex and of the thalamus included in Fig. 7.

EEG signals were down-sampled to 250 Hz, high-pass filtered at 0.1 Hz (Chebyshev II filter) and notch filtered at 50 Hz and its first harmonic (100 Hz). Channels located on the forehead and cheeks which mostly contribute to movement-related noise were discarded^72^, retaining thus 107 channels out of 128 (see SM-H). For each period, epochs with EEG signals exceeding 100 μV were automatically discarded; retained signals were visually inspected for the removal of artifacted epochs and noisy channels (rejected channels range: 3–24). Rejected signals were substituted with signals obtained via spline-interpolation^73^. Retained epochs of each session and period (pre- and post-) were concatenated and submitted to an Independent Component Analysis to remove ocular and/or muscular artifacts^74^. After the pre-processing, periods’ time length ranged from 3 to 5 min. Signals were finally re-referenced to the mastoids’average^75^ (reducing thus the number of channels to 105, see SM-H), and divided in 2 s non-overlapping epochs. Details about breathing signals processing and analyses are provided in SM-C.

### Statistical analysis procedures

Throughout the manuscript, except where otherwise stated, descriptive statistics are expressed as mean ± standard error. For all the EEG-derived features, the same statistical approach was applied. For each feature, t-tests with Statistical non Parametrical Mapping (SnPM) correction for statistic images^22,23^ between post- and pre-stimulation were conducted both for NS and SC. For each feature, comparisons between post-NS-stimulation and post-SC-stimulation were conducted after an appropriate normalization of the feature to its pre-stimulation value (SM-B). Differences between normalized features of post-NS and post-SC were assessed using SnPM (SM-I). SnPM was chosen to deal with the multiple testing issue which arises when considering simultaneous testing on either dense electrode arrays, voxels or between-hubs connectivities as it is a simple yet robust approach to control for type I statistical errors (i.e. rejection of a true null-hypothesis). Throughout the manuscript only p-values lower than 0.05 will be considered significant.

### Band spectral content evaluation

For each subject, session and period, a spectral analysis was conducted. The mean power spectrum density of each channel was evaluated applying a Hamming-windowed Fast Fourier Transform on 2 s epochs and averaging between them. Power density was evaluated for five bands of interest: delta (1–4 Hz), theta (4–8 Hz), alpha (8–12 Hz), beta (12–30 Hz) and gamma (30–45 Hz) averaging among the bins pertaining to the band and log-transforming the mean value.

Both for NS and SC, post-/pre-period differences were assessed for each band and electrode conducting paired t-tests; t-values significance was estimated based on SnPM.

For each session and band, the channel log-power in the post- was divided by the log-power in the pre-period (normalization). Normalized powers of the NS were compared to those related to the SC, performing for each band and electrode a t-test between the two conditions (SM-B). Significance levels were assessed applying SnPM correction on the obtained t-values.

### sLORETA in delta and theta bands

In line with our working hypothesis, all further analyses were conducted focusing on delta and theta bands. Sources spectral densities were estimated for each band by means of standardized low-resolution brain electromagnetic tomography (sLORETA) in the frequency domain^25,26^ for each 2 s epoch using sLORETA free software^26^. The mean cortical spectral density distribution of each band and period was obtained averaging among its epochs. sLORETA is a discrete three-dimensionally distributed, linear minimum norm inverse solution. The head model for the inverse solution takes advantage of the electric potential leadfield computed using the Boundary Element Method developed by Fuchs and colleagues^76^ applied to the MNI152 template^77^. The solution space is restricted to cortical gray matter, extracted using Talairach atlas^78^. The cortical volume is segmented in 6239 voxels with a spatial resolution of 5 mm; each sLORETA cortical image thus represent the standardized current density at each voxel in Montreal Neurological Institute (MNI) space. Both anatomical structures and Brodmann areas coordinates are reported in MNI space with correction to Talairach space^79^. Before the application of sLORETA, electrode positions were registered to the MNI152 space using sLORETA free software^26^.

Differences in cortical power densities distributions between post- and pre-periods were assessed for each band using t-tests with SnPM correction, both for NS and SC. For each band and subject, the mean cortical source power density distribution of post-period was then divided by its corresponding pre-stimulation distribution. Significant differences between normalized post-NS and post-SC were assessed applying t-tests with SnPM correction.

### Cortical hubs selection and evaluation

Eight cortical hubs were selected (see Fig. 3A) including areas of interest hypothesized by Fontanini and Bower^3^, and structures pertaining to the DMN^17^ (6 hubs):

- OFC (BAs 10, 11, 47)
- mPFC (BA 9)
- ACC (BAs 24, 32, 33)
- left-OA and right-OA, including the uncus, the anterior portion of the parahippocampal gyrus and the entorhinal cortex (BAs 27, 28, 34)
- PCC/PC (BAs 7, 23, 31)

Note that for each hub, only the main associated BAs are indicated.

Two additional structures (parahippocampal gyrus, BAs 35,36, and Primary Somatosensory Areas, BAs 3,1,2, PSA) were selected to clarify whether the cortical effects of NS could be either ascribable primarily to a trigeminal involvement or to the olfactory epithelium stimulation, which would in turn activate the olfactory system^49^. Should the former mechanism be involved, we would expect a relevant activation of PSA after the NS^50^, whereas a relevant activation of the parahippocampal gyrus would point to the latter, given the well-assessed relationship between respiration (and olfactory system) and hippocampus^13,14,51^.

For each hub, t-values related to voxels showing a significant post-NS/post-SC difference were retained (both for delta and theta). The t-values series were submitted to a one-way ANOVA with *hubs* as an eight-levels between-factor. F-significance was assessed using a randomization approach^80^ (SM-EF). Planned post-hocs between each hub and PSA were then conducted (paired t-tests with SnPM correction, SM-FI).

For each hub of interest, the mean spectral density value for each band, subject, session and period, was estimated averaging among its voxels. It is worth underlining that we choose to analyze average activations of large hubs (each composed by hundreds of voxels, i.e. with volumes in the order of cm^3^) so as to avoid possible biases related to the presence of however minimal source localization errors, which would instead become a major issue if conducting the analyses considering clusters of few voxels.

### Granger causality between cortical hubs in the frequency domain

Each EEG epoch was submitted to a time domain sLORETA^26^ extracting the standardized current density time-course of each voxel. For each hub, the mean current density time-course was estimated averaging among its voxels: six signals were obtained for each subject, session, period and epoch. Granger Causality (GC) analyses in the frequency domain were conducted taking advantage of the Causal Connectivity Toolbox functions^81^. Epochs standing the stationarity test^82^ were retained for the analyses (on average 80% of epochs *per*-period retained). The epoch model order was estimated using Akaike Information Criterion^83^. For each period, the median of its epochs model orders was chosen as the representative order of the period. The 95^th^ percentile of subjects’ model orders was taken as the overall order^84^ (20 samples, corresponding to 80 ms). GCs in the frequency domain^27^ between all couples of hubs were then evaluated for each epoch. Let *x* and *y* be two wide-sense stationary time-series, the Granger Causality $G{C}_{y\to x}$, is a measure of the contribution of the past of *y* time-series to the prediction of the present value of *x*; this measure is compared to the contribution of the *x* past in the prediction of its own present value. The GC has a spectral decomposition and as such, inferences about causal relations can be limited to a specific frequency or band of interest:^27^ the spectral GC is the proportion of power of *x* at the frequency (band) of interest deriving from its interaction with *y* (see SM-J). Let us express the spectral GC at *ω* frequency as $G{C}_{Y\to X}(\omega )$. We estimated the GC for two specific frequency band (delta and theta); considering a generic band $B=[{\omega }_{1},{\omega }_{2}]$, its GC is expressed by:

1 $$G{C}_{Y\to X}(B)={({\omega }_{2}-{\omega }_{1})}^{-1}{\int }_{{\omega }_{1}}^{{\omega }_{2}}G{C}_{Y\to X}(\omega )d\omega $$

The validity of the model order choice was a posteriori verified estimating the model consistency (percentage of the data correlation structure explained by the model)^85^. Consistency values higher than 75% are considered satisfactory^85^ (all periods had mean consistencies of 76% or above). For each couple of hubs, period and band, the mean GCs were estimated averaging between epochs values. For each band, couple of hubs, session and GCs series of values, a comparison between post- and pre-stimulation (be it NS or SC) was conducted (t-tests with SnPM). GCs related to post-NS were compared to those of the post-SC (after dividing each by its value in the pre-period): significance levels were assessed using t-tests with SnPM.

### Cortical hubs net Causal Flow of Information

For each period, band and hub, the net Causal Flow of Information (CFI) was estimated as the sum of the outcoming and incoming information flows:

2 $$CF{I}_{i}(B)=\sum _{j=1}^{n}G{C}_{i\to j}(B)-\sum _{j=1}^{n}G{C}_{j\to i}(B),j\ne i$$

In (2) ‘*i’* is the selected hub, ‘*n’* denotes the total number of hubs. For each session, band and hub, differences between post- and pre-stimulation CFIs were assessed based on t-tests with SnPM approach. For each session, band and cortical hubs couple ‘(*i*, *j*)’, the GC of the post-period was normalized to that related to the pre-period:

3 $$nG{C}_{po(i\to j)}(B)=2G{C}_{po(i\to j)}(B)/(|G{C}_{po(i\to j)}(B)|+|G{C}_{pr(i\to j)}(B)|)$$

In (3) ‘*po’* and ‘*pr’* refer to post-period and pre-period. The normalized CFI was then estimated as follows (*‘i’* is the hub):

4 $$nCF{I}_{i}(B)=\sum _{j=1}^{n}nG{C}_{i\to j}(B)-\sum _{j=1}^{n}nG{C}_{j\to i}(B),j\ne i$$

Significant differences in normalized-CFI values between post-NS and post-SC were assessed again using t-tests with SnPM.

### Psychometric assessment

At the end of each session, since our hypothesis predicts a modulation of the cortical activity (with a prevalent frontal involvement) driven by the nasal epithelium mechanical stimulation, a selection of psychometric tests specifically designed for evaluating integrated frontal functions was administered to identify cognitive effects of the stimulation: Digit Span Forward and Backward (subsets of Wechsler Memory Scale^20^) to evaluate numeric working-memory function and Corsi block test^21^ to evaluate visuospatial working memory.

We verified whether the NS induced as after-effects states of altered consciousness comparable to those described as a state-effects of meditation, using the Phenomenology of Consciousness Inventory (PCI)^19^. PCI is a retrospective self-report questionnaire allowing a phenomenological quantification of subjective experience. PCI assesses 12 dimensions of consciousness: altered experience; positive affect, negative affect, attention, imagery, self-awareness, state of awareness, internal dialogue, rationality, volitional control, memory and arousal.

For each psychometric scale or when appropriate, sub-scale, differences between post-NS and post-SC were assessed using paired t-tests. For each test, the t-value was extracted, and its significance was estimated based on a permutation test (1000 permutations, SM-E)^80^. An overall False Discovery Rate < 0.05 on the psychometric tests was obtained applying Benjamini and Hochberg’s correction^86^.

### Correlations between psychometric differences (NS versus SC) and differences in cortical hubs power densities

Psychometric tests showing significantly different scores between NS and SC were correlated to the band-power densities of those hubs showing a significantly different activation between post-NS and post-SC (delta and theta bands). Hubs’ band power densities in the post- (either NS or SC) where divided by those of the corresponding pre-period and the difference between NS and SC was computed for each subject and band. Those differences where correlated with psychometric scores differences between NS and SC (Pearson’s correlation). For each psychometric test, r-value significance was assessed using SnPM, rendering the test free of any gaussianity assumption and dealing at the same time with the multiple comparisons issue.

### Data availability

The datasets generated during and/or analysed during the current study are available from the corresponding author on reasonable request.

## Electronic supplementary material

supplementary material
