# Supplementary material for: Ultra-slow mechanical stimulation of olfactory epithelium modulates consciousness by slowing cerebral rhythms in humans
Source: Sci Rep. 2018 Apr 26;8:6581. doi: 10.1038/s41598-018-24924-9 (PMC5919905; doi:10.1038/s41598-018-24924-9)
Supplement: Supplementary file 1 — supplementary material [file 41598_2018_24924_MOESM1_ESM.pdf]

Ultra-slow mechanical stimulation of olfactory epithelium modulates consciousness by slowing cerebral rhythms in humans

Piarulli A[1,2], Zaccaro A[1], Laurino M[3], Menicucci D[4], De Vito A[5], Bruschini L[1,5], Berrettini S[1,5], Bergamasco M[6], Laureys S[2], Gemignani A[1,3,5,*]

1 Department of Surgical, Medical, Molecular and Critical Area Pathology, University of Pisa, Via Roma 65, 56126, Pisa, Italy.

2 Coma Science Group, GIGA Research Center, University and University Hospital of Liège, Avenue de l'Hôpital 11, 4000, Liège, Belgium.

3 Clinical Physiology Institute, National Research Council (CNR), Via Giuseppe Moruzzi 1, 56127, Pisa, Italy.

4 Department of Translational Research and New Technologies in Medicine and Surgery, University of Pisa, Via Risorgimento 36, 56126, Pisa, Italy.

5 Azienda Ospedaliero-Universitaria Pisana (University Hospital, AOUP), Via Paradisa 2, 56124, Pisa, Italy.

6 PERCRO Laboratory, TECIP Institute, Sant'Anna School of Advanced Studies, Via Alamanni 13B, 56010, Ghezzano, Pisa, Italy.

** Corresponding author, Professor Angelo Gemignani, Department of Surgical, Medical, Molecular and Critical Area Pathology, Via Roma 65, 56126, Pisa, University of Pisa, Italy.*

E-mail: angelo.gemignani@unipi.it

Phone Number: +39050992658

SECTION A: air delivery device setup and mode of operation and polygraphic system overview.

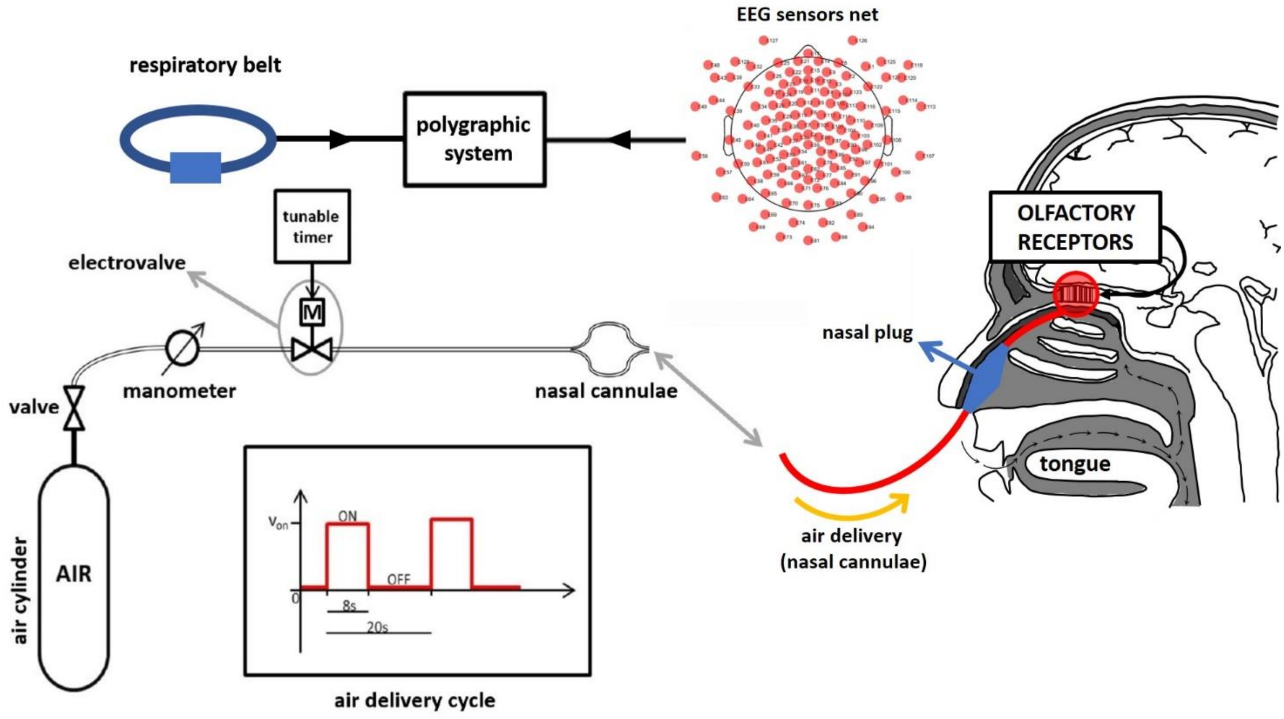

Figure A.The figure depicts a schematic representation both of the air delivery device, and of the polygraphic system. Note the presence of nasal plugs filling the whole nostrils holes. As mentioned in the main manuscript the nasal plug is provided with a pass-through hole for the positioning of the nasal cannula. In the embedded box, the air-delivery cycle is represented

SECTION B: flowchart of EEG features statistical analyses

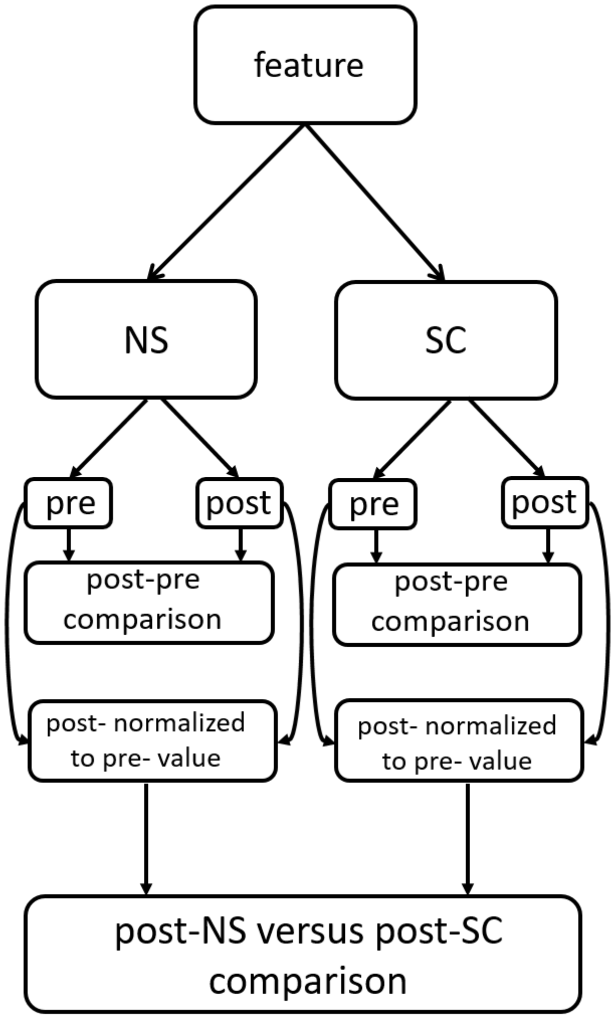

Figure B. Each EEG-derived feature was collected both for the nasal stimulation (NS) and the sham control (SC) sessions. For each session, values related to pre- and post- periods were estimated. The first set of comparisons were intra-session (post- versus pre- values). The second set of analyses concerned the between-sessions comparisons (post-NS versus post-SC). Before conducting each comparison, the feature value was normalized to its corresponding pre-period value. Normalization, except were otherwise stated, was always obtained as the ratio between post- and pre- values. The between-session comparisons were performed after a proper normalization to avoid possible confounding effects stemming from differences in electrodes impedances values between the two sessions. Such differences usually lead to non-physiological differences in EEG voltages, strongly biasing the results of between-sessions comparisons.

SECTION C: analysis of breathing rhythms

The subjects' breathing signals were acquired using a piezo-resistive respiratory belt placed on their abdomen. Signals were acquired for both conditions (NS-SC) and periods (pre-post). Respiratory signals were acquired for each subject, session and period, (sampling rate 500Hz, online band-pass filtering 0.01-20Hz). Three subjects were discarded from the analysis as their respiratory signals were largely affected by noise making it impossible to extract their breathing rhythm. For each retained subject and period, the signal was divided in 60s epochs with a 50% overlap between contiguous ones. Each epoch was submitted to a Hamming-windowed Fast Fourier Transform. Mean spectrum density as a function of frequency were obtained for each period averaging between epochs pertaining to the period itself, its peak denoting the breathing frequency as shown in the exemplary figure presented below.

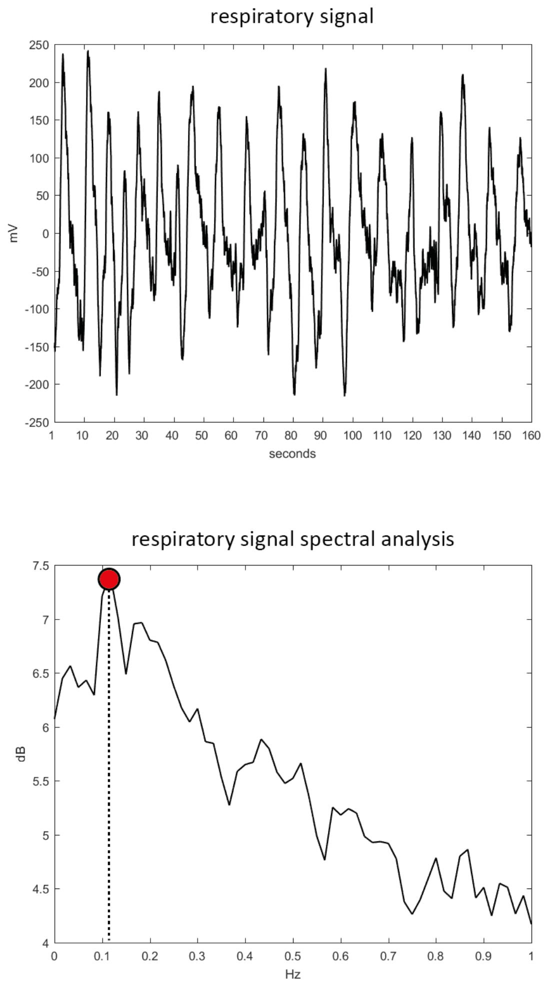

Figure C1. An exemplary case of breathing signal (first plot) and the related mean spectral density (second plot) is presented. The peak frequency of the mean spectrum density is of 0.1167Hz (red dot) corresponding to a breathing rhythm of 7 breaths/minute.

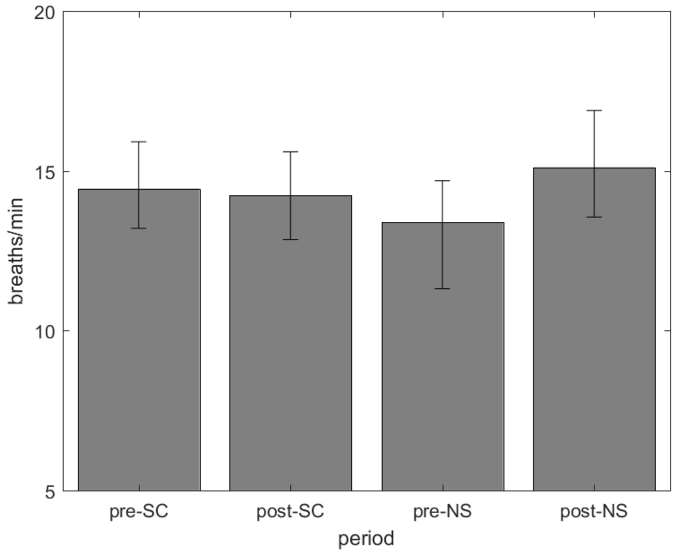

Figure C2. In the present figure breathing rhythms of the four periods are presented (mean ± standard error).

The series of breathing rhythms (9 subjects, 4 conditions) was submitted to a repeated measures ANOVA with *condition* (NS-SC) and *period* (pre-post) as within factors. The significance of F-values related to *condition*, *period* and their interaction was estimated on the basis of 1000 randomization of the original set of values (36, i.e. 4 per-subject) under the null-hypothesis of no significant effect for any factor. For each randomization, the F-values of *condition*, *period* and *condition*period,* were collected obtaining thus for each of them, the F-values distribution under the null- hypothesis (i.e. obtained by chance). For each factor, the related p-value was obtained as the ratio between the F-values of the null-distribution exceeding the real one and the number of randomizations (Manly, 2006). No significant effect was found for any of the three factors of the ANOVA, see TABLE C.

References

Manly, B. F. J. *Randomization, bootstrap and Monte Carlo methods in biology, volume 70* (CRC Press, 2006).

TABLE C1. For each within-factor, degrees of freedom, F-value, F-value threshold for significance at $p < 0.05$ and the obtained p-value are reported.

factor	Df	F-value	F-threshold for p < 0.05	p-value
Condition (NS-SC)	1	0.04	5.03	p = 0.40
Period (pre-post)	1	1.64	5.07	p = 0.13
Condition*Period	1	4.39	5.33	p = 0.58

For the sake of completeness, even if the omnibus tests were not significant, planned post-hoc were performed: i) NS post- versus pre- ii) SC post- versus pre- and iii) post-NS versus post-SC. For each comparison the t-value was extracted. 500 random permutations of the original dataset were performed under the null-hypothesis of no between-condition effect. Under the null, for each subject the value for condition1 could be attributed to condition2 and vice-versa. For each permutation the corresponding t-value was extracted (in absolute value for two-tails significance assessment). We obtained thus the t-values distribution under the null-hypothesis. The original t-value significance was obtained as the ratio between the number of randomly generated t-values exceeding the absolute value of the real one and the number of randomizations. This number was set to 500 as with 9 subjects and two conditions the maximum number of possible different randomizations would have been $2^9=512$.

TABLE C2. For each post-hoc, the t-value, the t-value significance threshold for $p < 0.05$ and the estimated p-value are reported.

**comparison**	**t-value**	**|t-value| threshold for p < 0.05**	**p-value**
NS post-pre	1.71	2.57	p = 0.55
SC post-pre	-0.89	2.80	p = 0.18
NS-post vs SC-post	0.57	1.88	p = 0.68

SECTION D: SC post-pre comparison

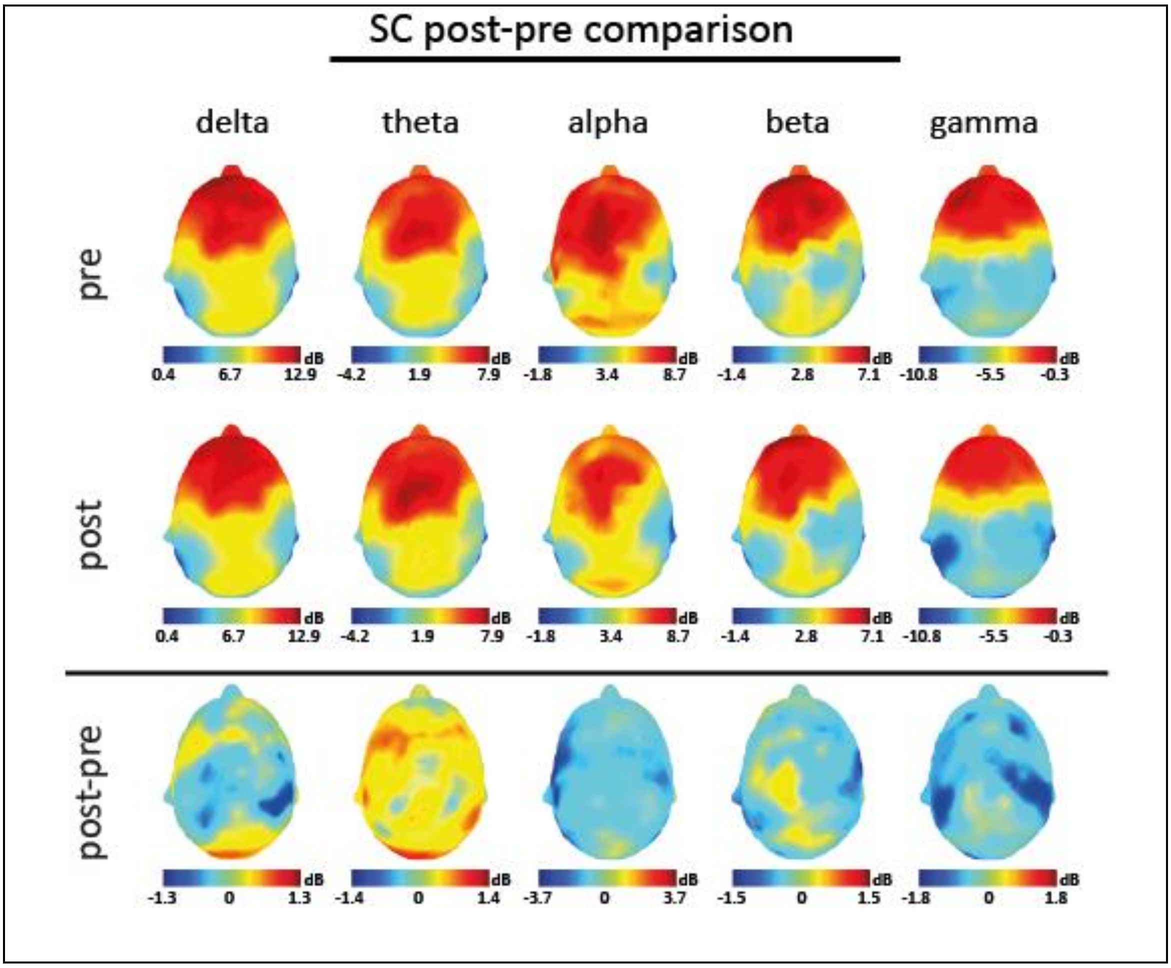

Figure D. Topographical distributions of log-transformed power in the five bands of interest are presented for the sham session. First row refers to the pre-SC, second row to the post-SC and third row to the comparison between post- and pre-SC. Note that for pre- and post-sham maps the same scale is used to help the reader in the interpretation of the comparisons. No significant difference between post- and pre-sham is apparent for any of the considered bands (see third row).

SECTION E: Randomization tests

- No assumption about distributions normality or homoscedasticity.
- Null-hypothesis: no effect or treatments/conditions…. (i.e. under the null the value associated with a subject/feature is independent from the condition).
- Any statistic can be used depending on the dataset organization and on the formulated null-hypothesis.
- The output of the chosen statistic is not compared to its tabled distributions but rather to outputs obtained calculating the statistics of choice on datasets generated by the repeated randomization of data across groups (treatments/conditions).
- Significance is obtained as the ratio between the number of outputs (in absolute value) generated from the randomizations higher than the real output (taken in absolute value) and the total number of randomizations.

As an example, let us consider the one-way ANOVA with *hubs* as an eight-levels between-factor.

- The F-value statistics is extracted.
- We formulate the null-hypothesis of no significant *hubs*-effect.
- Under the null-hypothesis values related to hubs can be relabeled randomly (i.e. a value from hub1 attributed to hub3, a value from hub4 to hub8 and so on).
- One thousand random relabeling are performed collecting their F-values.
- The significance of the original F-value is obtained as the ratio between the randomly generated F-values exceeding the real one, and the total number of relabeling.

SECTION F: comparing the stimulation effects on the selected hubs to those related to the primary sensory area

Table F1. Results of the one-way ANOVA with *hub* as between-subject effect are presented together with the result of the planned post-hocs (each hub versus PSA) both for delta and theta bands. For each statistical test, relevant information are reported (ANOVA: F and p values; paired t-tests: relationships between mean values, t and p-values). Note that the F-values significance was evaluated using the randomization approach described in the main text and in SM-E; t-values significance was evaluated for each band using the SnPM approach described both in the main text and more in depth in SM-H.

Band	ANOVA, *hub- effect*	post-hocs versus PSA					
		OFC	mPFC	ACC	OA	PC/PCC	PH
**Delta**	F = 104.7<br>**p < 0.001**	**OFC>PSA**<br>t = 5.52,<br>**p < 0.001**	**mPFC<PSA**<br>t = -11.77,<br>**p < 0.001**	ACC>PSA<br>t = 0.13,<br>p = 1;	**OA>PSA**<br>t = 5.59,<br>**p < 0.001**	**PC/PCC>PSA**<br>t = 8.43,<br>**p < 0.001**	PH>PSA<br>t = 0.55<br>p = 1
**Theta**	F = 238.9,<br>**p < 0.001**	**OFC>PSA**<br>t = 26.82,<br>**p < 0.001**	**mPFC>PSA**<br>t = 11.59,<br>**p < 0.001**	**ACC>PSA**<br>t = 21.16<br>**p < 0.001**	**OA>PSA**<br>t = 22.48,<br>**p < 0.001**	**PC/PCC>PSA**<br>t = 3.73,<br>**p < 0.001**	**PH>PSA**<br>t = 15.20<br>**p < 0.001**

Table F2. Significance threshold obtained using randomization test for assessing F-values significance and significance thresholds obtained for t-values after applying SnPM correction for statistic images are reported

Band	F-values' significance thresholds			t-values' significance thresholds		
	p < 0.05	p < 0.01	p < 0.001	p < 0.05	p < 0.01	p < 0.001
**Delta**	2.20	2.98	4.04	2.71	3.19	3.46
**Theta**	2.12	2.81	3.81	2.62	3.07	3.56

SECTION G: Causal Flow of Information, confirmatory analyses

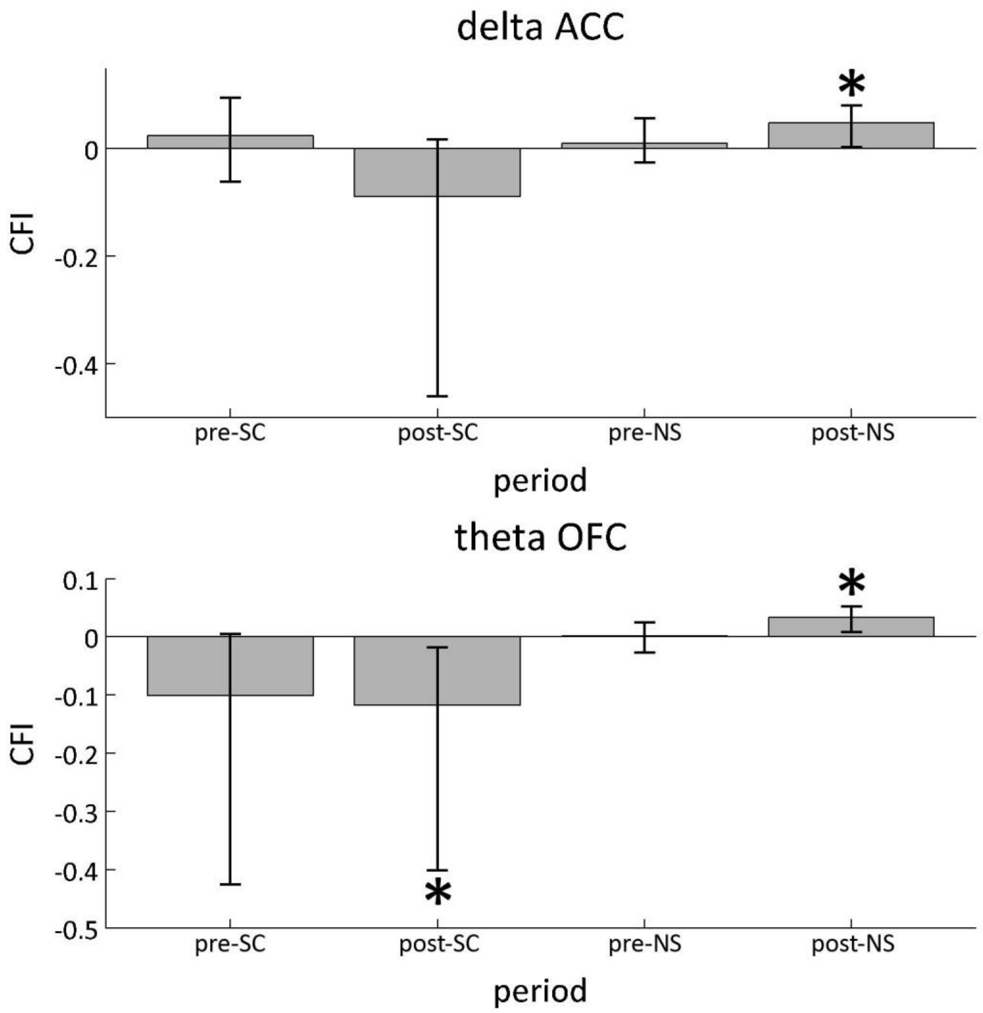

Figure G. Descriptive statistics of net Casual Flow of Information for the ACC in delta band and for the OFC in theta band are presented for the four periods (pre- and post-SC, pre- and post-NS). Grey bars represent the mean values of the distributions while error-bars their 95% confidence interval. Black asterisks denote significance at $p < 0.05$.

SECTION H: Retained electrodes

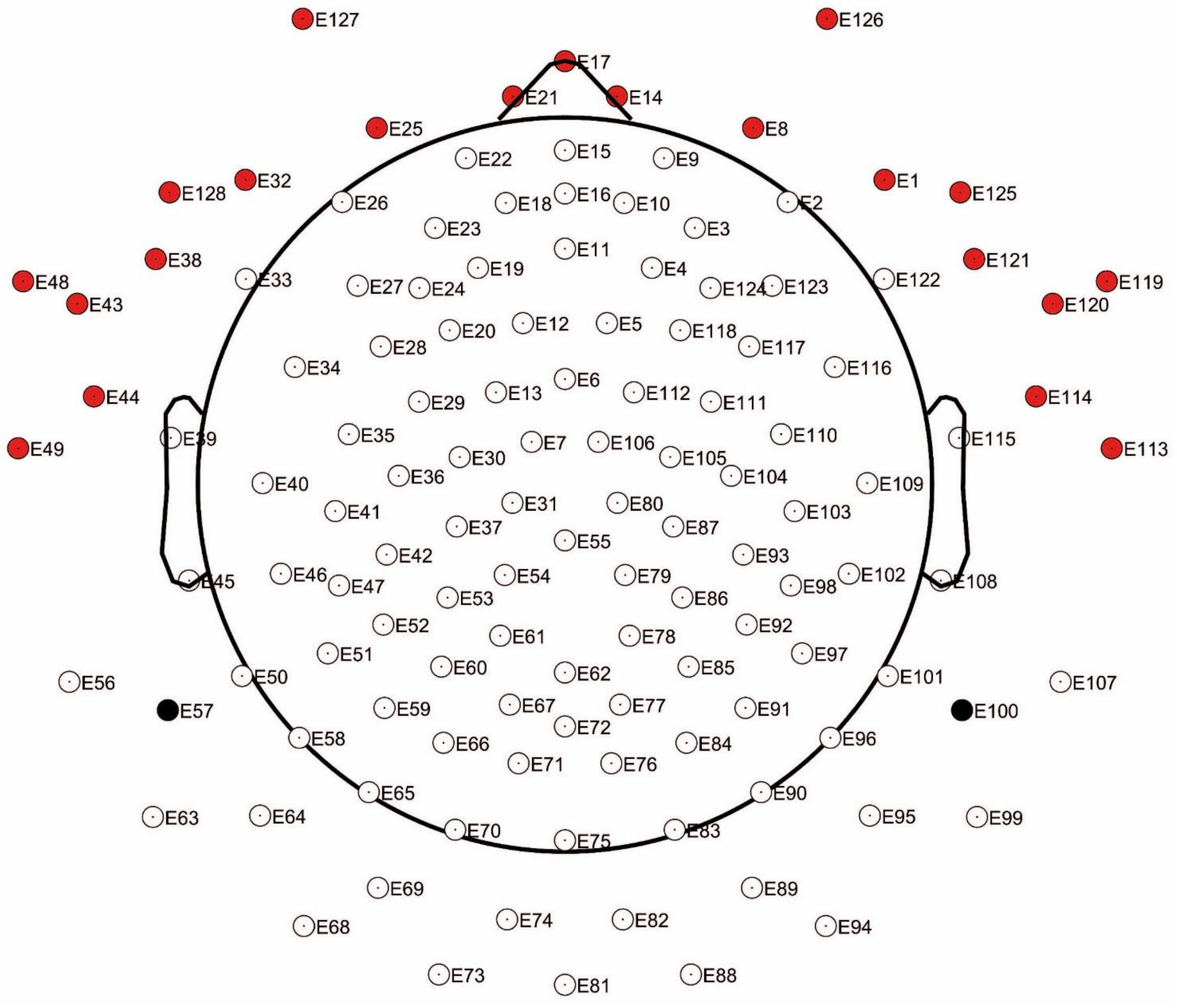

Figure H. Electrodes locations.

Of the 128 electrodes of the EGI sensor net, 21 located on the cheeks and forehead were removed as they mostly contributed to movement-related noise (red dots in Figure H). The 107 retained channels were then re-referenced to the mastoids' (black dots in Figure H) average reducing the analyzed electrodes to 105.

SECTION I: Statistical non-Parametric Mapping, SnPM.

Let us briefly introduce the basis and rationale of SnPM: assume without loss of generality to have collected an EEG feature for each electrode in two different conditions. For each electrode, a paired t-test between the conditions is conducted and its t-value is retained. As the test is applied to multiple electrodes, a single-threshold SnPM procedure is used to assess the significance of each t-test, considering the multiple comparison issue. Let us consider the null-hypothesis of no significant condition-effect: under this null-hypothesis the labeling of the collected feature can be changed randomly (i.e. a feature estimated for condition 1 can be assigned to condition 2 and vice-versa). Based on this assumption, 1000 random relabeling are made, and for each of them, the t-value related to each single comparison is extracted. For each relabeling, only the maximum t-value (in absolute value, for two-tailed significance assessment) among simultaneous comparisons is retained. In this manner, the maximum t-value distribution under the null-hypothesis of no significant condition-effect is extracted. The significance of each original t-value is then estimated as the ratio between the number of t-values of the null-distribution exceeding the original t-value (in absolute value) and the number of relabeling.

SECTION J: Granger Causality in the frequency domain

Herein we briefly rehearse the mathematical basis and hypotheses underlying the spectral domain Granger Causality: let *x* and *y* be two wide-sense stationary time-series, the Granger Causality $GC_{y \to x}$, is a measure of the contribution of the past of *y* time-series to the prediction of the present value of *x*; this measure is compared to the extent of the contribution of the *x* past in the prediction of its own present value. More in deep, the comparison is made between the unrestricted and the restricted models. The unrestricted model is expressed by the following equation:

$$\begin{bmatrix} x(t) \\ y(t) \end{bmatrix} = \sum_{k=1}^{n} a_k \begin{bmatrix} x(t-kt_0) \\ y(t-kt_0) \end{bmatrix} + \begin{bmatrix} e_x(t) \\ e_y(t) \end{bmatrix} \quad (1).$$

In (1) a_k are the *2x2* matrices of the model coefficients, *n* is the model order, $e_x(t)$ and $e_y(t)$ are the residuals. The restricted model is mathematically described by:

$$x(t) = \sum_{k=1}^{n} b_k x(t-kt_0) + \hat{e}_x(t) \quad (2).$$

In equation (2), b_k are the model coefficients, *n* is the model order and $\hat{e}_x(t)$ is the residual. Please note that for both models t_0 represents the time-lag between consecutive samples. Coefficients (i.e. a_k and b_k) and residuals (i.e. $e_x(t)$, $e_y(t)$ and $\hat{e}_x(t)$ can be estimated using linear autoregressive methods such as ordinary least squares. GC in the time-domain is thus defined as the logarithm of the ratio between the residual variance in the restricted model and that in the unrestricted one:

$$GC_{y \to x} = \log_e \left[\frac{\mathrm{var}(\hat{e}_x)}{\mathrm{var}(e_x)} \right] \quad (3).$$

As demonstrated by **Geweke (1982)**, the GC has a spectral decomposition and based on such decomposition, inferences about causal relations can be limited to a specific frequency or band of interest: the spectral GC is the proportion of power of *x* at the frequency (or band) of interest deriving from its interaction with *y*. Equation (1) can then be rewritten in Fourier space as:

$$A(\omega) \begin{bmatrix} X(\omega) \\ Y(\omega) \end{bmatrix} = \begin{bmatrix} E_X(\omega) \\ E_Y(\omega) \end{bmatrix} \quad (4).$$

Upper case letters denote the Fourier transforms. For the sake of clarity let us express *A(ω)* as:

$$A(\omega) = \begin{bmatrix} A_{xx}(\omega) & A_{xy}(\omega) \\ A_{yx}(\omega) & A_{yy}(\omega) \end{bmatrix} \quad (5).$$

The unrestricted model regression coefficients can be then expressed as:

$$A(\omega) = I - \sum_{k=1}^{n} a_k e^{-i\omega k} \quad (6).$$

In equation (6), *I* is the identity matrix. Let us now rewrite equation (4) as:

$$\begin{bmatrix} X(\omega) \\ Y(\omega) \end{bmatrix} = \begin{bmatrix} H_{xx}(\omega) & H_{xy}(\omega) \\ H_{yx}(\omega) & H_{yy}(\omega) \end{bmatrix} \begin{bmatrix} E_x(\omega) \\ E_y(\omega) \end{bmatrix} \quad (7).$$

In (7), *H* is the transfer matrix and the following equation holds $H=A^{-1}$. The spectral matrix *S* can then be written as:

$$S(\omega) = \langle X(\omega) X^*(\omega) \rangle = \langle H(\omega) \Sigma H^*(\omega) \rangle \quad (8).$$

In (8) Σ is the covariance matrix of residuals in the unrestricted regression which can be expressed as:

$$\Sigma = \begin{bmatrix} \sigma_{xx} & \sigma_{xy} \\ \sigma_{yx} & \sigma_{yy} \end{bmatrix} = \mathrm{cov} \begin{bmatrix} e_x(t) \\ e_y(t) \end{bmatrix} \quad (9).$$

Let us now apply the transformation:

$$y \to y - \frac{\sigma_{yx}}{\sigma_{xx}} \quad (10),$$

which diagonalizes Σ, leaving GC invariant (**Barrett *et al.*, 2012**). Then the spectral GC at a frequency ω can be expressed as:

$$GC_{Y \to X}(\omega) = \log_e \left(\frac{S_{xx}(\omega)}{H_{xx}(\omega) \sigma_{xx} H_{xx}^*(\omega)} \right) \quad (11).$$

Combining equation (8) with (10) the following equation can be obtained:

$$S_{xx}(\omega) = H_{xx}(\omega) \sigma_{xx} H_{xx}^*(\omega) + H_{xy}(\omega) \sigma_{yy} H_{xy}^*(\omega) \quad (12).$$

Let us finally assume that we want to estimate the GC for a specific frequency band, be it $B = [\omega_1, \omega_2]$, the following equation can be applied:

$$GC_{Y \to X}(B) = (\omega_2 - \omega_1)^{-1} \int_{\omega_1}^{\omega_2} GC_{Y \to X}(\omega) d(\omega) \quad (13).$$

References

Geweke, J. Measurement of linear dependence and feedback between multiple time series. *J. Am. Stat. Assoc.* **77**, 304-313 (1982).

Barrett, A. B. *et al.* Granger causality analysis of steady-state electroencephalographic signals during propofol-induced anaesthesia. *Plos One* **7**, e29072; 10.1371/journal.pone.0029072 (2012).
